# Thermal Generalism Shapes Tolerance to Extreme Heatwaves in Estuarine Fishes

**DOI:** 10.1111/gcb.71143

**Published:** 2026-10-19

**Authors:** Angus Mitchell, David J. Booth, Sean D. Connell, Timothy Ravasi, Ivan Nagelkerken

**Affiliations:** ^1^ Southern Seas Ecology Laboratories, School of Biological Sciences Adelaide University Adelaide South Australia Australia; ^2^ School of Life Sciences University of Technology Sydney Ultimo New South Wales Australia; ^3^ Marine Climate Change Unit Okinawa Institute of Science and Technology (OIST) Onna‐son Okinawa Japan; ^4^ College of Science and Engineering James Cook University Townsville Queensland Australia

**Keywords:** climate variability hypothesis, climatic extremes, coastal fisheries, ecophysiology, estuarine fishes, marine heatwaves, oxidative stress, thermal tolerance

## Abstract

Climate change is widely expected to push many taxa beyond their biological limits, yet some populations persist in environments that routinely experience climatic extremes. This raises a fundamental question: does their thermal tolerance reflect species‐level thermal generalism (an evolved capacity to maintain performance across a broad thermal range) or population‐specific acclimation to local conditions (phenotypic plasticity)? Distinguishing between these alternatives is key for predicting whether tolerance to climatic extremes will generalise across species distributions or remain restricted to heat‐tolerant populations. To test these competing hypotheses, we quantified physiological performance (at cellular and organismal levels) in three economically important estuarine fish ( *Acanthopagrus australis* , *Sillago ciliata* and *Rhabdosargus sarba* ) collected from 20 sites across 15 estuaries along a ~1000‐km latitudinal gradient spanning their core and poleward range edges. Sites differed in thermal variability, including some that intermittently exceeded the severest end‐of‐century climate projections. We then experimentally exposed two of the fish species from low and high thermal variability sites to moderate (+2°C, 14 days) or extreme (+4°C, 7 days) simulated marine heatwave events, assessing 12 physiological and behavioural proxies. All three species collected in situ maintained comparable physiological performance across sites of contrasting thermal variability. Both experimental species, each collected from a single high‐ and low‐variability site, tolerated heatwave conditions regardless of thermal history or heatwave intensity, supporting species‐level thermal generalism rather than population‐specific acclimation. In the absence of heatwave conditions, *S. ciliata* from high‐variability sites also showed steeper allometric scaling and faster growth at 26 versus 24°C experimental control temperatures, suggesting this species may benefit from the warmer baseline temperatures projected under gradual climate change. Combined, our findings support thermal generalism as the primary driver of tolerance in estuarine fishes to climatic extremes, with important implications for the fisheries they support under climate change.

## Graphical abstract

Competing predictions for how estuarine fish respond to marine heatwaves. Under population‐specific acclimation (left), the performance of fish from low‐variability sites decline as intensity increases while high‐variability fish are buffered by prior exposure. Under thermal generalism (right), both maintain performance regardless of origin. Our results support species‐level thermal generalism.

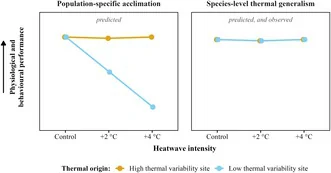

## 1 Introduction

Climatic extremes are increasing in frequency, intensity and duration across Earth's biomes as a consequence of anthropogenic climate change (IPCC 2023; Frölicher et al. 2018; Perkins‐Kirkpatrick and Lewis 2020). Extreme events such as floods, droughts, storms and heatwaves are now recognised as dominant drivers of ecological change (Fischer et al. 2021), often exerting impacts that exceed those associated with gradual shifts in mean climatic conditions (Ummenhofer and Meehl 2017).

In aquatic systems, climatic extremes can rapidly restructure communities, disrupt ecosystem functioning, and compromise ecosystem services, including fisheries productivity (Wernberg et al. 2016; Smale et al. 2019; Cheung et al. 2021). Vulnerability to climatic extremes is particularly high in aquatic systems because the physiological performance of aquatic organisms is tightly constrained by temperature‐dependent metabolism and oxygen availability, and because extreme events can rapidly develop over spatial and temporal scales that limit behavioural avoidance (Pörtner and Farrell 2008; Pörtner and Peck 2010; Smale et al. 2019). Marine heatwaves have emerged as a pervasive climatic extreme capable of driving mass mortality, transient range shifts and persistent ecosystem change across coastal and open‐ocean systems (Oliver et al. 2018; Smale et al. 2019). Consequently, fishes are widely considered highly susceptible to climatic extremes, with elevated temperatures predicted to reduce aerobic scope, disrupt energy allocation and alter behaviour, ultimately constraining growth, reproduction and survival (Franklin and Seebacher 2009; Pörtner and Peck 2010; Johansen and Jones 2011; Nagelkerken et al. 2023). A substantial body of work on the thermal physiology of temperate and polar fishes has documented sensitivity to warming at these taxa's upper thermal limits (Pörtner and Peck 2010; Verberk et al. 2026). However, much of the empirical basis for broader vulnerability under marine heatwave conditions specifically has been derived from studies of coral reef fishes (Nagelkerken et al. 2023), which evolved under comparatively stable environmental conditions and often exhibit narrow thermal safety margins (Johansen and Jones 2011; Johansen et al. 2024). While these studies provide important insight into climate sensitivity in environmentally stable aquatic systems, their dominance in the heatwave literature raises the possibility that vulnerability frameworks may underrepresent taxa that routinely experience high natural climate variability and have potentially evolved broad thermal tolerance as a consequence.

This underrepresentation in the literature is particularly relevant in estuarine ecosystems, which are among the most physically variable environments on Earth (Elliott and Whitfield 2011; Whitfield et al. 2012; Baumann and Smith 2018) and are becoming increasingly susceptible to climate change (Scanes et al. 2020; Khojasteh et al. 2025). Estuaries experience large, rapid and recurrent fluctuations in temperature, salinity, pH, and dissolved oxygen across diel, seasonal and episodic timescales, frequently reaching or exceeding conditions projected for many open‐ocean ecosystems later this century (Baumann and Smith 2018; Scanes et al. 2020; Khojasteh et al. 2025). Despite this extreme abiotic variability, estuarine fish communities persist and support highly productive fisheries (Gillanders et al. 2011; Koehn et al. 2011), and some estuarine species may even benefit from ocean warming (Coleman et al. 2019; Tan et al. 2024). Estuarine systems therefore provide a powerful setting to evaluate whether chronic exposure to climatic variability confers tolerance to climatic extremes—either through population‐level acclimation and adaptation driven by local thermal history, or through the inherent thermal generalism of species that have evolved in chronically variable coastal environments.

The *climate variability hypothesis* predicts that organisms persisting in highly variable environments may evolve or maintain broad physiological tolerance ranges rather than narrow performance optima (Janzen 1967; Gaston 2009; Sunday et al. 2014). This prediction can be evaluated at two scales. At the species level, broadly distributed taxa that have persistently occupied variable environments throughout their evolutionary history may exhibit inherent thermal generalism across populations—potentially arising through mechanisms such as phenotypic plasticity, genetic adaptation or both. At the population level, the *climate variability hypothesis* predicts that populations chronically exposed to greater thermal variability should show enhanced tolerance to climate extremes relative to conspecifics from low variability environments, reflecting local acclimation to thermal variability. These two scales of prediction are not mutually exclusive but generate distinct empirical expectations—thermal generalism predicts equivalent tolerance across all populations, whereas at the population‐level the *climate variability hypothesis* predicts a performance advantage specifically in fish from high‐variability sites under stressful conditions. Across taxa, long‐term exposure to environmental variability has been linked to broader thermal tolerance limits and reduced sensitivity to acute stressors (Stillman 2003; Gunderson and Stillman 2015), yet empirical tests that explicitly distinguish between these two scales of prediction in fishes remain limited.

Here, we address this gap by combining a large‐scale field study spanning a 1000‐km latitudinal gradient across eastern Australia with a controlled simulated marine heatwave experiment. We focused on three economically important estuarine fishes: the yellowfin bream ( *Acanthopagrus australis* ), sand whiting ( *Sillago ciliata* ) and tarwhine ( *Rhabdosargus sarba* ), which are broadly distributed across subtropical and temperate estuaries and persist across a wide range of abiotic conditions. Their broad distributional ranges and persistence across highly variable abiotic conditions make them strong candidates for thermal generalism—the capacity to maintain physiological performance across a wide thermal range regardless of local thermal history. We then used a simulated heatwave experiment to assess whether physiological tolerance inferred from the field translated into tolerance under either a 14‐day +2°C heatwave event or a 7‐day +4°C heatwave event, and whether responses depended on thermal history or the intensity–duration structure of the heatwave.

Specifically, we tested two competing hypotheses about the basis of thermal tolerance, and a third about heatwave structure: (1) a *thermal generalism hypothesis*, predicting that behavioural and physiological performance metrics, including total protein content, antioxidant capacity, oxidative damage and length‐weight relationships would be maintained across populations from high‐ and low thermal variability estuaries and under heatwave conditions irrespective of thermal history, reflecting species‐level tolerance shaped by evolutionary history in chronically variable environments; (2) the alternative *pre‐exposure hypothesis*, predicting that fishes from high‐variability estuaries would show elevated or maintained performance under heatwaves relative to those from low‐variability estuaries, consistent with population‐level acclimation driven by local thermal history; and (3) an *event‐profile hypothesis*, predicting that extreme high‐intensity heatwaves would impose greater costs in low‐variability estuary populations than moderate, longer heatwaves of equivalent cumulative thermal exposure. Combined, this approach allowed us to distinguish between species‐level thermal generalism and population‐level thermal history as drivers of climatic tolerance in estuarine fishes, while simultaneously testing whether heatwave structure determines short‐term performance outcomes across broad biogeographic scales.

## 2 Materials and Methods

### 2.1 Study Sites

To test the tolerance of economically important fishes to climatic extremes, we sampled estuarine fishes from 20 sites across 15 estuary systems, encompassing tidal rivers, coastal lagoons (including intermittently closed and open lakes and lagoons; ICOLLs), and marine‐influenced embayments spanning ~1000 km of Australia's east coast (~28°–38° S; Figure 1b). Estuaries along this coastline are characterised by pronounced environmental variability and can experience sustained periods of elevated temperature that exceed adjacent coastal waters (Figure 1a).

**FIGURE 1:**
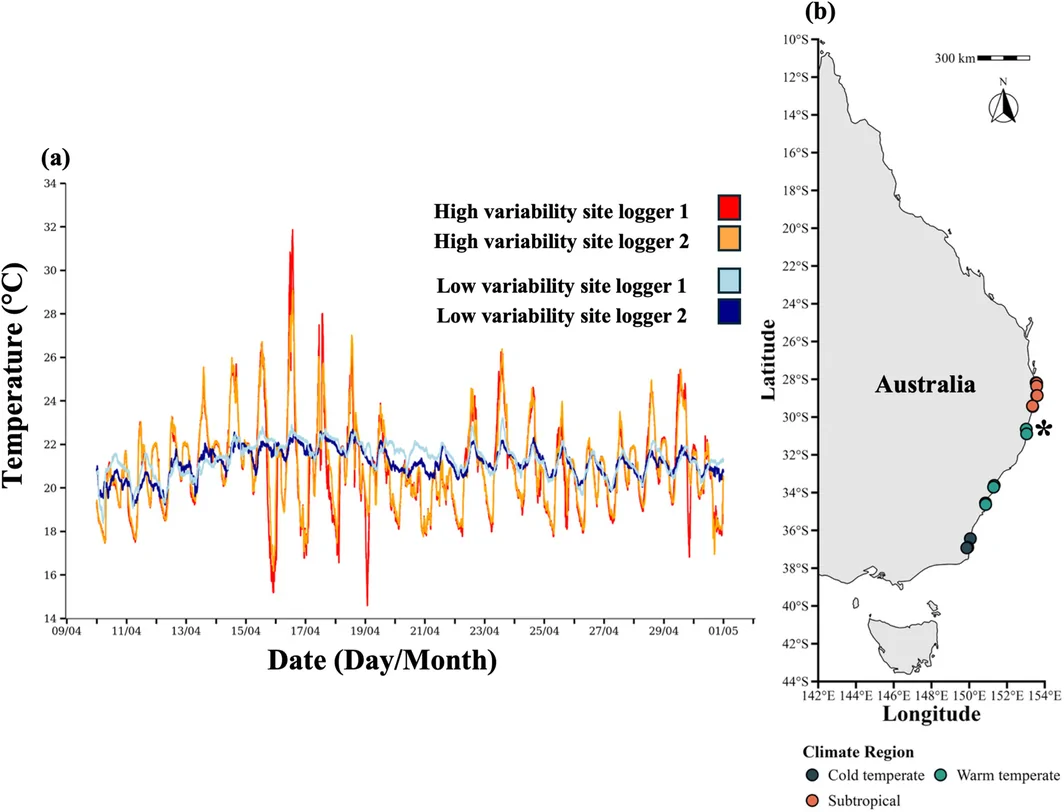
(a) High‐resolution temperature time series (°C) recorded by replicate loggers at the high‐variability site (HVS; red/orange lines) and low‐variability site (LVS; light and dark blue lines) between 9 April and 1 May 2024. The high‐variability site showed greater diel and short‐term temperature fluctuations, including more frequent excursions beyond preferred temperature ranges, relative to the low‐variability site. Juvenile yellowfin bream ( *Acanthopagrus australis* ) and sand whiting ( *Sillago ciliata* ) collected from these sites were used in the simulated marine heatwave experiment. (b) Estuary sampling sites along the east coast of Australia spanning approximately 1000 km and three biogeographic regions: Cold temperate (dark blue), warm temperate (light blue) and subtropical (orange). *Denotes high‐ and low‐variability sites for fish used in the simulated heatwave experiment. Sampling estuaries included tidal rivers, coastal lagoons (including intermittently closed and open lakes) and marine‐influenced embayments. Map lines delineate the study areas and do not necessarily depict accepted national boundaries.

Estuaries sampled included the Tweed River (Tweed River entrance and Wommin Lagoon; 28.18° S, 153.54° E; 28.21° S, 153.57° E), Cudgera Creek (Cudgera Creek and Christies Creek; 28.36° S, 153.57° E), the Richmond River (South Ballina reach and Prospect Lake; 28.86° S, 153.57° E; 28.86° S, 153.60° E), the Clarence River (Whiting Bay Inlet and Iluka Bay; 29.44° S, 153.36° E; 29.41° S, 153.35° E), Deep Creek (Valla; 30.60° S, 153.01° E), the Nambucca River (Stuart Island; 30.64° S, 153.02° E), Back Creek (South West Rocks; 30.89° S, 153.04° E), Careel Bay (33.60° S, 151.32° E), Narrabeen Lagoon (33.70° S, 151.29° E), Lake Illawarra (34.55° S, 150.86° E), the Minnamurra River (34.63° S, 150.87° E), the Bermagui River (river entrance and Rose Bay; 36.44° S, 150.06° E; 36.43° S, 150.05° E), Merimbula Lake (36.90° S, 149.91° E), Back Lagoon (36.90° S, 149.92° E) and Pambula Lake (36.93° S, 149.87° E).

At the Tweed, Richmond, Bermagui and Clarence River systems, as well as Cudgera Creek, we sampled paired habitats (e.g., river main channels and adjacent lagoons or embayments) to capture fine‐scale heterogeneity in thermal and oxygen conditions within individual estuaries.

Across all biogeographic regions, water temperatures during summer at study sites frequently exceeded long‐term regional summer averages (Tables S1, S2, and S4). In subtropical estuaries, where mean maximum summer temperatures are typically ~27°C, in situ measurements during our field sampling reached ~37°C. In warm temperate estuaries, where average summer temperatures are ~23°C–24°C, water temperatures exceeded 32°C (Figure 1a; Tables S1 and S2). In cold‐temperate estuaries, where mean summer temperatures are generally ~22°C, temperatures reached ~26°C. High‐ and low‐variability sites were distributed across all three biogeographic regions and are classified in Table S1.

### 2.2 Temperature Monitoring and Water Chemistry

To characterise site‐level thermal variability, water temperature, salinity, dissolved oxygen (DO), and pH were measured at each site during fish collections using calibrated handheld probes (Ohaus StarterPen Salinity Meter IC‐ST20; StarterPen Dual Channel pH/Conductivity Water Analysis Meter; Portable Dissolved Oxygen Analyzer IC‐LH‐D701). Measurements were taken 3–5 times per collection period per site, and all sensors were calibrated prior to use according to manufacturer’s instructions.

Temperature loggers were deployed at a subset of sites during sampling periods to characterise short‐term thermal variability at depths of 0.5–1.5 m. Logger deployment duration varied among sites and sampling periods, reflecting the available time during fish collection at each site. During the January 2024 sampling period, logger deployments ranged from approximately 3.9 to 50.1 h of continuous temperature recording across sites; in March 2024, deployments were shorter, ranging from 0.5 to 4.6 h. During both sampling periods, Onset HOBO Pendant temperature loggers were deployed at each site on sampling days and programmed to record temperature at 1‐min intervals. Temperature loggers were deployed during fish collection periods to characterise the thermal environment experienced by sampled fish, with short‐term deployments reflecting conditions at the time of collection. The consistency between short‐term deployments and month‐long records at Narrabeen Lagoon and Careel Bay provides confidence that these data accurately captured the relative thermal regimes of HVS and LVS sites.

Continuous temperature logger data were collected at all sites during both sampling periods, except for Wommin Lagoon, Cudgera and Christies Creeks during the January sampling period, where point‐based temperature measurements collected during fish collection were used to characterise ambient thermal conditions. These point measurements were included in summaries of site conditions but were not used to calculate thermal variability metrics derived from continuous logger data. For estuaries with continuous logger record, the following thermal metrics were calculated for each site and sampling period using all available observations: mean temperature, minimum temperature, maximum temperature, standard deviation and temperature range (maximum minus minimum temperature).

### 2.3 Thermal Regime Comparison Between High‐ and Low‐Variability Estuarine Sites

During the January 2024 sampling period, mean water temperatures at high‐variability sites (HVS) ranged from 27.7°C to 29.7°C, exceeding those at low‐variability sites (LVS; 25.3°C–26.2°C). Thermal variability was substantially greater at HVS, with standard deviations of 3.4°C–4.2°C, compared with 1.1°C–2.0°C at LVS. Correspondingly, temperature ranges were large at HVS (12.9°C–14.5°C) and narrower at LVS (3.6°C–8.2°C). Minimum temperatures were similar between site types (HVS: 22.4°C–23.6°C; LVS: 21.6°C–23.8°C), but maximum temperatures diverged strongly, reaching 36.5°C–37.8°C at HVS compared with ≤ 30.1°C at all LVS (Table S2). In March, mean temperatures ranged from 24.7°C to 29.5°C at HVS and from 20.4°C to 26.5°C at LVS. Standard deviations at HVS varied from 0.16°C to 2.7°C, with several systems exhibiting elevated variability relative to LVS, which generally showed low dispersion (0.07°C–0.65°C). Temperature ranges at HVS reached up to 9.1°C, compared with ≤ 2.8°C at all LVS, and most LVS (9 of 12) had ranges below 2°C. Minimum temperatures overlapped between site types (HVS: 20.7°C–27.9°C; LVS: 19.6°C–25.8°C), whereas maximum temperatures were generally higher at HVS, reaching 34.7°C, compared with ≤ 26.9°C at LVS (Table S2). All other abiotic variables measured (pH, salinity and dissolved oxygen) were comparable between HVS and LVS during January–March 2024 (Table S1).

### 2.4 Study Species and Collections

We collected three commercially important estuarine fishes: yellowfin bream ( *Acanthopagrus australis* , *n* = 168), sand whiting (*Sillago ciliata, n* = 351) and tarwhine ( *Rhabdosargus sarba* , *n* = 44; Table S3). Species were selected because they co‐occur across thermally benign and extreme estuaries, support valuable recreational and commercial fisheries (Koehn et al. 2011; Gillanders et al. 2011), and span distinct trophic niches and life‐history strategies. All three are broadly distributed across subtropical and temperate eastern Australia, persist across a wide range of abiotic conditions, and exhibit relatively broad preferred temperature ranges, making them strong a priori candidates for thermal generalism.

*A. australis* is an omnivorous, euryhaline sparid occupying estuaries, coastal rivers, lagoons, and nearshore marine habitats along eastern Australia (~19°–38° S), with preferred temperatures of 20.2°C–26.7°C (Froese and Pauly 2020). *S. ciliata* is a benthic invertivore with one of the broadest latitudinal ranges of any estuarine fish on the continent (~10°–41° S), inhabiting shallow estuaries, sandy bays and surf zones, with preferred temperatures of 21.2°C–27.4°C (Froese and Pauly 2020). *R. sarba* is an omnivorous sparid common across subtropical and temperate Australia, with juveniles using estuaries and lagoons as nursery habitats and preferred temperatures of 21.9°C–29.0°C (Froese and Pauly 2020). The persistence of all three species across thermally heterogeneous estuarine landscapes, including estuaries that intermittently experience temperatures well beyond their reported thermal optima, is consistent with the broad thermal tolerance expected of thermal generalists.

Fish were collected using a 10‐m seine net (2‐m drop) between 19 and 31 January and 4 and 18 March 2024. Field sampling during January 2024 followed a north‐to‐south progression to capture subtropical and warm temperate estuaries during their local summer thermal maximum. The March 2024 sampling period followed a south‐to‐north progression, prioritising southern cold‐temperate estuaries at their seasonal thermal peak before resampling northern estuaries to increase species and site replication. Following capture, fish were transferred to 50‐L buckets of site water, euthanised immediately by rapid brain spiking, and measured for total length (mm) and wet weight (g) before freezing at −20°C. Fish were then transferred to the University of Adelaide and stored at −80°C until processing for total protein content, antioxidant capacity, and oxidative damage assays.

### 2.5 Simulated Heatwave Experiment

#### 2.5.1 Study Sites and Species

To test the heatwave tolerance of two commercially important estuarine fishes, yellowfin bream ( *Acanthopagrus australis* ) and sand whiting ( *Sillago ciliata* ), we selected one high‐variability site (HVS) and one low‐variability site (LVS) at which both species co‐occurred. The HVS, Narrabeen Lagoon (33.7027° S, 151.3018° E), is an intermittently closed and open lagoon (ICOLL) subject to rapid fluctuations in temperature (Figure 1a). The LVS, Careel Bay (33.6050° S, 151.3202° E), is a permanently open estuary characterised by more stable daily temperature conditions.

#### 2.5.2 Microclimate Characterisation of Study Sites

To quantify local thermal regimes at the HVS and LVS, we deployed two Onset HOBO Pendant temperature loggers at each site: at 33.7030° S, 151.3010° E and 33.7113° S, 151.2979° E at the HVS (Narrabeen Lagoon), and at 33.6202° S, 151.3211° E and 33.6011° S, 151.3162° E at the LVS (Careel Bay). Temperature was recorded at 1‐min intervals from 9 April to 1–2 May 2024 (Figure 1a). Thermal variability was quantified as the mean daily thermal range (daily maximum minus minimum temperature), the standard deviation of temperature, and the mean absolute change between consecutive hourly mean temperatures (°C·h^−1^). Metrics were calculated separately for each logger and summarised at the site level (Table S6).

#### 2.5.3 Month‐Long Characterisation of Thermal Regimes at High‐ and Low‐Variability Sites

At the HVS (Narrabeen Lagoon) and LVS (Careel Bay) used for the simulated heatwave experiment, month‐long logger deployments confirmed substantially greater thermal variability at the HVS than at the LVS. Mean water temperatures were comparable between site types (20.8°C–21.3°C; Table S6). However, the standard deviation of temperature was 169%–210% higher at the HVS than at the LVS (1.91–2.08 vs. 0.67°C–0.71°C, respectively), and mean daily thermal ranges were 222%–298% greater (6.18–6.77 vs. 1.70°C–1.92°C). Mean hourly rates of temperature change were 4.1–4.8 times higher at the HVS than at the LVS (0.59–0.65 vs. 0.14°C·h^−1^; Table S6).

#### 2.5.4 Fish Collection and Experimental Setup

Juvenile *S. ciliata* and *A. australis* were collected between 26 and 30 March 2024 from the LVS (Careel Bay; −33.604980, 151.320170) and HVS (Narrabeen Lagoon; −33.702736, 151.301779) at depths of 0.5–1.5 m using barrier nets. Fish were transported to the Sydney Institute of Marine Science and housed in 100‐L ambient holding tanks (5–15 individuals per tank, separated by origin) at ambient water temperatures (~22°C–23°C), reflecting conditions at the time of collection. Fish were then randomly assigned to one of six temperature treatments (*n* = 10 per treatment; Table S5) across two climate‐controlled rooms. Each treatment was replicated in 22‐L transparent flow‐through tanks (39 × 28 × 28 cm; *n* = 120 tanks), fitted with PVC pipe shelter (10 cm long, 6.5 cm diameter) and bilateral flow‐through via two 0.5‐cm outflow holes 2 cm below the surface. Water temperature was maintained within ±0.5°C of setpoints via solenoid feedback systems at a flow rate of ~200 mL·min^−1^. Each tank housed a single fish serving as the experimental unit (*n* = 1 fish per tank; 120 tanks total).

The experiment commenced on 10 April 2024, with each fish measured for total length (±0.01 mm) and wet weight (±0.01 g) at the start. Mean (±SE) starting sizes were 88.23 ± 3.24 mm and 14.77 ± 1.61 g for *A. australis* , and 70.33 ± 2.53 mm and 3.39 ± 0.35 g for *S. ciliata* . Fish were acclimated by ramping temperature at 1°C·day^−1^ to origin‐specific control temperatures (LVS: 24°C; HVS: 26°C), with fish assigned to higher temperatures introduced earlier to standardise acclimation duration. Heatwave fish were held at control temperatures for 5 days before ramping at +1°C·day^−1^ to treatment maxima. No mortality occurred during acclimation. All fish were fed *ad libitum* twice daily: *S. ciliata* received frozen adult Artemia, and *A. australis* a mixed diet of frozen Artemia and AquaOne Tropical Bits consistent with their omnivorous diet (O'Connor and Booth 2021).

Control fish were maintained at origin‐specific baseline temperatures (LVS: 24°C; HVS: 26°C) for the full duration of the experiment and sampled alongside heatwave‐treated fish at the end of the exposure period. All behavioural and physiological metrics, including hepatosomatic index, were assessed across control and heatwave treatment groups.

#### 2.5.5 Experimental Rationale

Fishes from the high‐variability site routinely experience rapid and large thermal fluctuations, including short‐term spikes that already reach or exceed projected near‐future summer conditions (Figure 1a). Given this thermal exposure history, we set the control temperature for high‐variability site fish to reflect a warmer baseline consistent with the conditions they regularly experience (26°C). In contrast, fish from the low‐variability site do not encounter highly variable temperature fluctuations (Figure 1a) and were assigned a control temperature reflecting present‐day summer conditions (24°C). This origin‐specific baseline allowed us to test whether behavioural and physiological responses reflect prior environmental history rather than short‐term acclimation, while acknowledging that differences between site types could partly reflect their different control temperatures rather than population‐specific adaptation.

From these controls, we established two heatwave treatments designed to be equivalent in cumulative thermal intensity (28°C·days, calculated as anomaly × duration) but different in their profile. The moderate heatwave was +2°C for 14 days (2°C × 14 days = 28°C·days), approximating the average heatwave observed in the Sydney bioregion (~34° S) between 1982 and 2024 (Table S4). The extreme heatwave was +4°C for 7 days (4°C × 7 days = 28°C·days), representing more intense events projected to occur more frequently by 2100 (Oliver et al. 2018). For low‐variability site fish, this equated to 26°C (moderate heatwave) and 28°C (extreme heatwave), while for high‐variability site fish it equated to 28°C (moderate heatwave) and 30°C (extreme heatwave). By holding cumulative intensity constant while varying magnitude and duration, our experimental design isolated whether behavioural and physiological responses are governed primarily by the total heat dose or by the profile of the event (longer–moderate vs. shorter–extreme).

Our experimental design therefore allowed us to test whether responses to marine heatwaves reflect prior exposure to thermal variability (*pre‐exposure hypothesis*) and whether physiological and behavioural performance is governed primarily by cumulative thermal exposure or by the intensity–duration profile of heatwaves (*event‐profile hypothesis*).

### 2.6 Video Observations of Behaviour

On Day 5 of each heatwave treatment, behavioural responses were quantified from 30‐min video recordings (GoPro Hero 7 Silver, 30 cm from tank), following a 3‐min settling‐in period, as in Mitchell et al. (2023a), across three sequential 10‐min periods. Behavioural responses were selected to reflect ecologically relevant performance across key fitness‐related domains: activity was used as an indicator of energy expenditure, shelter use as a proxy for predation risk management, feeding initiation time and bite rate as measures of foraging performance and appetite, and flight initiation distance as a measure of predator avoidance capacity. Combined, these behavioural metrics provide a broad assessment of whether heatwave conditions alter behaviours likely to influence survival, energy acquisition and fitness in the wild (Mitchell et al. 2023a; O'Connor and Booth 2021). During Period 1, we quantified activity (proportion of time in sustained swimming) and shelter use (proportion of time within the PVC shelter). During Period 2, ~1 g of frozen *Artemia* suspended in 25 mL seawater was introduced via syringe, and we recorded feeding initiation time (latency to first bite, s) and bite rate (bites·min^−1^) over the first 5 min. During Period 3, boldness and predator avoidance were assessed via a flight initiation distance (FID) test, in which a metal ruler (30 × 3 cm) was slowly lowered above the fish to simulate a looming stimulus, and the distance (cm) at onset of escape recorded. Non‐responses during feeding and FID trials (2.5% and 11.7% of trials, respectively) were distributed across treatment groups and origins with no systematic pattern.

#### 2.6.1 Completion of Simulated Heatwave Experiment

At the end of the exposure period (experimental Day 15 for the +4°C treatment and Day 20 for the +2°C treatment, with controls sampled alongside), fish were immediately euthanised by rapid brain spiking, flash‐frozen in liquid nitrogen and stored at −80°C until further processing for total protein content, antioxidant capacity, oxidative damage and hepatosomatic index, allowing direct comparison of physiological responses between field and experimental conditions.

#### 2.6.2 Fish Experimental Growth Rates

Fish total length (TL ± 0.01 mm) and wet weight (WW ± 0.01 g) were measured at the start and completion of the experimental exposure period for all fish. We then calculated the mean daily TL and WW growth rate (% initial size day^−1^) for each individual fish.

#### 2.6.3 Physiological Responses of Fish Exposed to Simulated Heatwaves and Field Conditions

All individuals collected across the 1000‐km gradient (*n* = 563; see Table S3) were measured for total length (TL) and wet weight (WW) to assess length–weight relationships and body condition. From this total, a subset of 294 fish was analysed for total protein content (TP), total antioxidant capacity (TAC) and lipid peroxidation (malondialdehyde; MDA). These metrics were selected as established proxies of cellular and organismal performance under thermal stress (Mitchell et al. 2023b; Missionário et al. 2024). Total protein content reflects the capacity for cellular repair and maintenance under stressful conditions. Total antioxidant capacity quantifies the ability to neutralise reactive oxygen species generated during thermal stress. Malondialdehyde concentration provides a direct measure of oxidative damage to cellular lipids. Length‐weight relationships and allometric scaling were included as indicators of somatic energy allocation, with the prediction that thermally stressed populations would show reduced mass gain per unit length relative to populations operating within their preferred thermal zone, a signal that would manifest as differences in allometric slope or intercept between HVS and LVS populations.

#### 2.6.4 Total Protein Content, Total Antioxidant Capacity and Oxidative Damage

We homogenised 50–100 mg of white muscle tissue (PBS; 10% w/v) on ice, and the homogenate was used to assess total protein content (TP), total antioxidant capacity (TAC) and malondialdehyde concentration (MDA, a marker of oxidative damage). Absorbance was measured using a Jenway 6405 spectrophotometer (Cole‐Parmer, Staffordshire). Assays followed manufacturer's protocols (Elabscience, Wuhan, China) with the following kits and detection wavelengths: TP (E‐BC‐K168‐S, 595 nm), TAC (E‐BC‐K136‐S, 520 nm) and MDA (E‐BC‐K025‐S, 532 nm). The TAC assay quantified the combined concentration of antioxidant macromolecules, small molecules and enzymes. Final concentrations were calculated using kit‐specific formulas:

$$\begin{array}{c} \mathrm{TP}\\ \left(g/L\right) \end{array}=\frac{\text{ODsample}-\text{ODblank}}{\text{ODstandard}-\text{ODblank}}\times \text{standard conc}.\left(0.563\;g/L\right)$$

$$\begin{array}{cc} \begin{array}{c} \mathrm{TAC}\\ \left(U/\mathrm{mg}\;\text{protein}\right) \end{array}\;=\; & \frac{\text{ODsample}-\text{ODcontrast}}{0.01}÷30\times \;\begin{array}{c} \text{volume of}\;\\ \text{homogenate} \end{array}\\ & ÷\frac{\text{protein conc}.}{\left(\text{mg protein}/\mathrm{mL}\right)} \end{array}$$

$$\begin{array}{cc} \begin{array}{c} \mathrm{MDA}\\ \left(\text{nmol}/\mathrm{mg}\;\text{protein}\right) \end{array}\;=\; & \frac{\text{ODsample}-\text{ODcontrast}}{\text{ODstandard}-\text{ODblank}}\times \begin{array}{c} \text{standard conc}.\\ \left(10\;\text{nmol}/\mathrm{mL}\right) \end{array}\\ & ÷\begin{array}{c} \text{protein conc}.\\ \left(\text{mg protein}/\mathrm{mL}\right) \end{array} \end{array}$$

#### 2.6.5 Hepatosomatic Index

Livers were dissected from fish at the end of the simulated heatwave experiment and weighed to the nearest 0.0001 g. The hepatosomatic index for each fish was calculated as liver weight relative to total fish wet weight and expressed as a percentage (Chellappa et al. 1995).

$$\text{Hepatosomatic index}=\frac{\text{weight of liver}\;\left(g\right)}{\mathrm{wet}\;\text{weight of fish}\;\left(g\right)}\times 100$$

#### 2.6.6 Length‐Weight Relationships

For field‐collected individuals, body condition was assessed using log–log linear models of the form ln(WW) = a + b·ln(TL), where a is the intercept and b the allometric scaling coefficient (Figure 2 displays these relationships on log_10_ axes). Differences in intercepts or slopes between HVS and LVS fish would indicate that populations differed in mass at a given length, providing a standardised measure of field body condition across species and regions.

**FIGURE 2:**
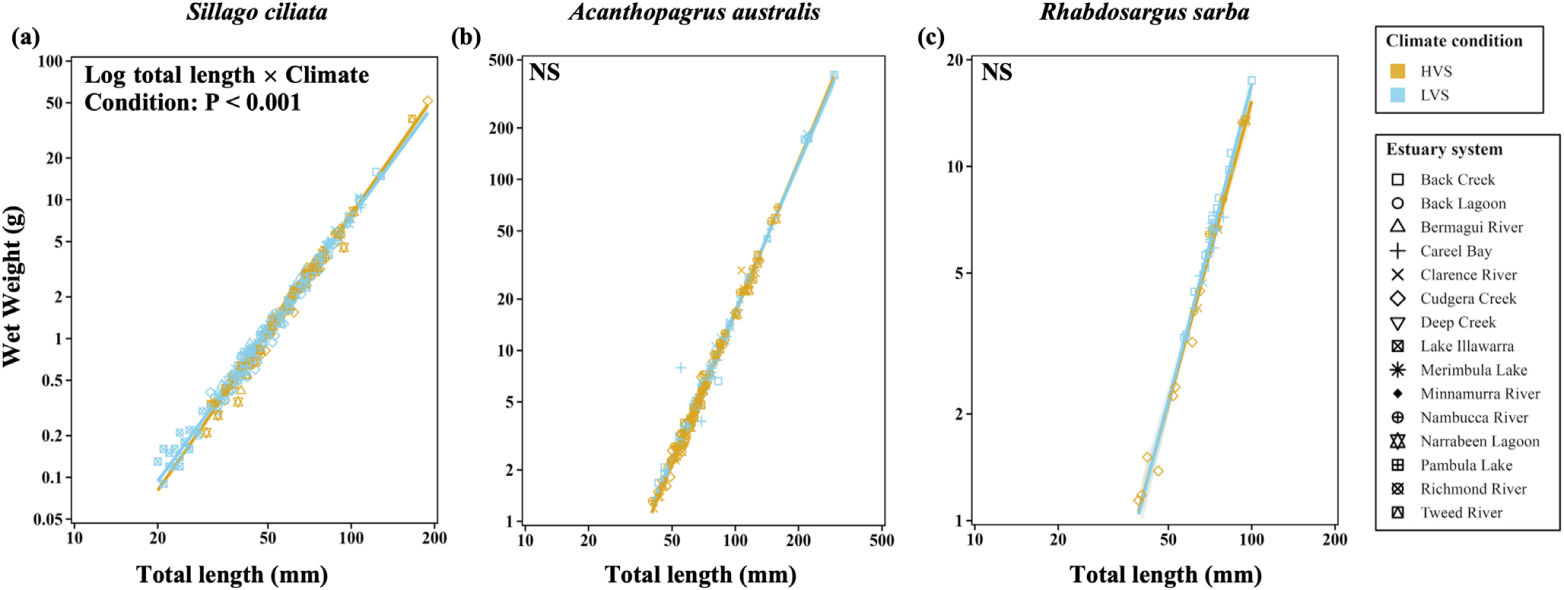
Length–weight relationships for three estuarine fish species collected from high‐variability (HVS, gold) and low‐variability (LVS, blue) estuaries spanning a ~1000‐km latitudinal gradient along the east coast of Australia. Panels show (a) *Sillago ciliata* (*n* = 351), (b) *Acanthopagrus australis* (*n* = 168) and (c) *Rhabdosargus sarba* (*n* = 44). Both axes are log_10_‐scaled and labelled with back‐transformed values. Symbols denote individual estuary systems. Lines show fitted regressions from linear mixed‐effects models (log_10_[wet weight] ~ log_10_[total length] × climate condition + (1 | estuary system)), with shaded bands indicating 95% confidence intervals. A significant interaction between log_10_ total length and climate condition was detected in *S. ciliata* (β = −0.125 ± 0.036 SE, *z* = −3.46, *p* < 0.001). No significant interaction was detected in *A. australis* (*p* = 0.160) or *R. sarba* (*p* = 0.418) denoted by “NS” in Panels b and c.

For fish used in the simulated heatwave experiment, body condition was assessed as size‐dependent change in mass over the trial, calculated as Δlog_10_(WW) = log_10_(WW_final)−log_10_(WW_start) and modelled against log_10_‐transformed starting total length (Δlog_10_(WW) = a + b·log_10_(TL_start)). This accounted for the influence of initial body size on growth potential and allowed us to test whether mass gain or loss differed among treatments while controlling for size‐dependent growth dynamics.

### 2.7 Statistical Analysis

All analyses were conducted in R version 4.4.0 (R Core Team 2024) and performed separately for each species to account for species‐specific size scaling and variance structure. For field‐collected fish, we analysed length–weight relationships and three physiological metrics—total protein (TP), total antioxidant capacity (TAC) and oxidative damage (MDA)—with all response variables natural‐log‐transformed prior to analysis (ln[WW] for wet weight; ln[x + 1] for physiological metrics) and ln‐transformed total length included as a covariate in all models to account for body‐size scaling. Length–weight relationships were modelled using an interaction between ln‐transformed total length and climate condition (ln[WW] ~ ln[TL] × climate condition + (1 | estuary system)) to test whether allometric scaling differed between site types, while physiological proxies were modelled additively (ln[proxy + 1] ~ ln[TL] + climate condition + (1|estuary system)), with size treated as a general scaling covariate rather than a context‐dependent response. In all models, HVS was set as the reference level so that the climate condition coefficient represents the contrast between LVS and HVS. Site identity was excluded as a random effect because sites were defined by their climate condition classification and its inclusion would have partially absorbed the fixed effect of primary interest; estuary system was therefore included as the sole random intercept to account for non‐independence of fish collected from the same estuary. Where random intercept variance was negligible, models were effectively equivalent to fixed‐effects regressions; this occurred in most physiological proxy models and is noted in Table S8. All models were fitted using the package glmmTMB (Brooks et al. 2017) with a Gaussian error distribution, assumptions were evaluated using simulation‐based residual diagnostics in DHARMa (Hartig 2024), and explanatory power was quantified using marginal (*R* ^2^m) and conditional (*R* ^2^c) coefficients of determination following Nakagawa and Schielzeth (2013), calculated with the MuMIn package (Bartoń 2025).

For the heatwave experiment, the effects of origin (high‐ vs. low‐variability site), heatwave treatment (control, +2°C, +4°C) and their interaction were tested separately for *A. australis* and *S. ciliata* using Gaussian generalised linear models (GLMs). Response variables included growth (total length and wet weight growth rates, % day^−1^), body condition scaling (Δlog₁₀[WW]), physiological proxies (TP, TAC, MDA and HSI) and behavioural metrics (activity, sheltering, feeding initiation time, flight initiation distance and bite rate). For body condition, log_10_‐transformed starting total length and its interactions with origin and treatment were included as covariates to account for size‐dependent growth dynamics. Model assumptions were evaluated using standard diagnostic plots. The significance of origin, treatment and their interaction was assessed using Type III tests with sum‐to‐zero contrasts (likelihood‐ratio tests for GLMs; *F*‐tests for the body condition model) in the car package (Fox and Weisberg 2019). Where the interaction was significant, Tukey‐adjusted pairwise comparisons were made among treatments within each origin and between origins within each treatment using emmeans (Lenth 2026). Model fit is reported as *R* ^2^ and adjusted *R* ^2^. Fish that died during the experiment (5.8%; Table S12) were excluded from growth and physiological analyses; their behaviour, recorded on Day 5 before any mortality occurred, was retained.

## 3 Results

### 3.1 Physiological Performance of Estuarine Fishes Across a Latitudinal Gradient

For *S. ciliata* , length–weight scaling differed between site types (Figure 2a; Tables S7 and S8): mass increased more steeply with length in fish from HVS than in fish from LVS (log(TL) × climate condition: *β* = −0.125 ± 0.036 SE, *p* < 0.001), such that LVS fish were marginally heavier than HVS fish at small sizes but lighter above ~67 mm TL. No equivalent difference in length–weight scaling was detected in *A. australis* or *R. sarba* (Tables S7 and S8).

For the three cellular proxies—total protein content, total antioxidant capacity and oxidative damage (MDA)—physiological performance was largely comparable between fish from HVS and LVS in all three species, irrespective of latitude (Tables S7 and S8). Climate condition had no significant effect on total protein content or total antioxidant capacity in any species (all *p* ≥ 0.18; Table S8), and these models explained little variation (*R* ^2^m = 0.005–0.05). The exception was oxidative damage in *R. sarba* , in which fish from LVS had higher MDA concentrations than those from HVS (*β* = 0.309 ± 0.111 SE, *p* = 0.005; *R* ^2^m = 0.24; Table S8). Climate condition did not affect MDA in *S. ciliata* (*p* = 0.95) or *A. australis* (*p* = 0.52), although MDA declined weakly with body size in *S. ciliata* (*β* = −0.152 ± 0.073 SE, *p* = 0.037).

### 3.2 Estuarine Fish Behavioural and Physiological Responses to Simulated Marine Heatwaves

Under extreme (+4°C for 7 days) and moderate (+2°C for 14 days) simulated heatwave conditions, neither *A. australis* nor *S. ciliata* showed significant changes in physiological responses (length growth, wet weight growth, total protein content, total antioxidant capacity, oxidative damage, hepatosomatic index and length–weight scaling; Tables S9 and S10) or behavioural responses (flight initiation distance, feeding initiation time, bite rate, activity and sheltering; Table S11) relative to control temperatures, irrespective of whether fish originated from LVS or HVS (all *p* ≥ 0.06; Figure 3, Figures S1–S3). The only exception was total length growth in *S. ciliata* , for which origin and heatwave treatment interacted (χ^2^ = 7.62, *p* = 0.022; Figure 3b; Table S10): fish from the HVS grew faster than those from the LVS under control temperatures (*p* = 0.020), but not under either heatwave treatment (*p* ≥ 0.10).

**FIGURE 3:**
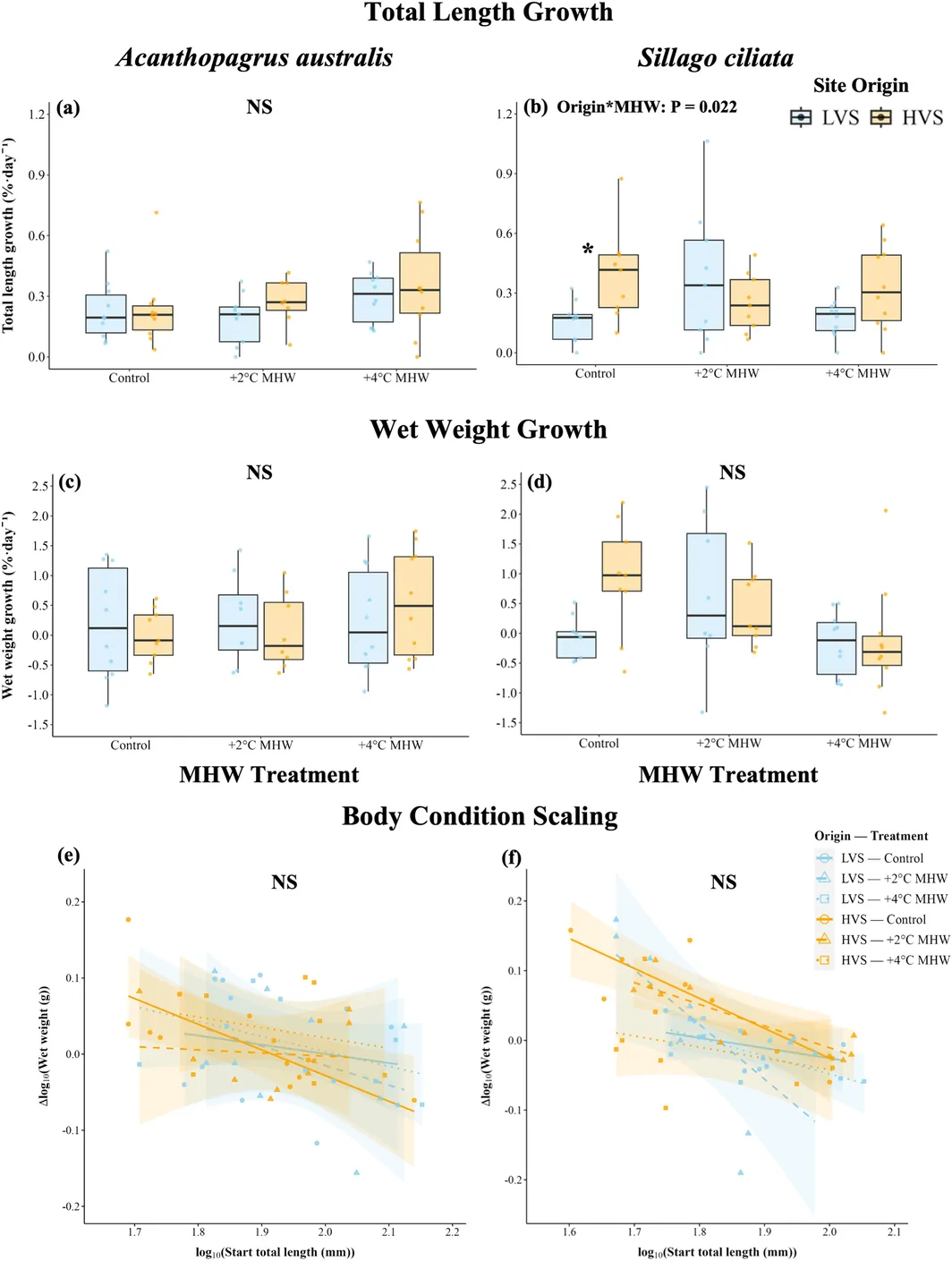
Estuarine fish growth responses to simulated marine heatwaves. (a–b) Total length growth rate (% day^−1^) and (c–d) wet weight growth rate (% day^−1^) for *Acanthopagrus australis* (yellowfin bream; panels a, c) and *Sillago ciliata* (sand whiting; panels b, d) originating from low‐variability (LVS; blue) and high‐variability (HVS; orange) estuarine sites across Control, +2°C MHW, and +4°C MHW treatments. Actual temperatures were 24°C (LVS Control), 26°C (LVS +2°C MHW; HVS Control), 28°C (LVS +4°C MHW; HVS +2°C MHW) and 30°C (HVS +4°C MHW). Boxplots show medians, interquartile ranges, and raw data points. (e–f) Size‐dependent growth relationships showing log_10_‐transformed change in wet weight (Δlog_10_ [wet weight]) plotted against log_10_‐transformed starting total length for *A. australis* (e) and *S. ciliata* (f). Regression lines and 95% confidence intervals are shown separately for each origin × treatment combination. ‘NS’ indicates no significant effects of treatment, origin or their interaction (Type III tests, *p* > 0.05). Asterisks denote significant differences between origins (LVS vs. HVS) under control temperatures only.

Mortality during the simulated heatwave experiment was low (5.8%) and distributed evenly across treatment groups and origins with no apparent systematic pattern (Table S12).

## 4 Discussion

Our findings provide evidence to support the *thermal generalism hypothesis*—all three estuarine fish species maintained comparable physiological performance across estuaries of contrasting thermal variability spanning a 1000‐km gradient, and the two species tested experimentally ( *A. australis* and *S. ciliata* ) maintained physiological and behavioural performance under simulated heatwave conditions, regardless of thermal origin or heatwave intensity and duration. Combined, the heatwave experiment and field results suggest that broad thermal tolerance is a fundamental characteristic of these estuarine fish species rather than a population‐specific response to local thermal history (Figure 4). While our data are consistent with the pre‐exposure hypothesis in that fish from both high‐ and low‐variability sites tolerated experimental conditions that already intermittently occur in these estuaries, the absence of a performance advantage in fish from high‐variability sites relative to those from low‐variability sites provides evidence against strong population‐level differentiation in thermal tolerance. Our results therefore support species‐level thermal generalism as the primary explanation for the tolerance we observed, suggesting that the evolutionary history of these species in chronically variable environments, rather than the thermal conditions experienced by individual populations, supports their broad climatic tolerance. This interpretation suggests that the *climate variability hypothesis* operates at a species scale in these taxa and is consistent with growing evidence that broadly distributed estuarine fishes are inherently buffered against thermal extremes that may threaten less eurythermal taxa (Coleman et al. 2019; Gunton et al. 2025; Sunday et al. 2014).

**FIGURE 4:**
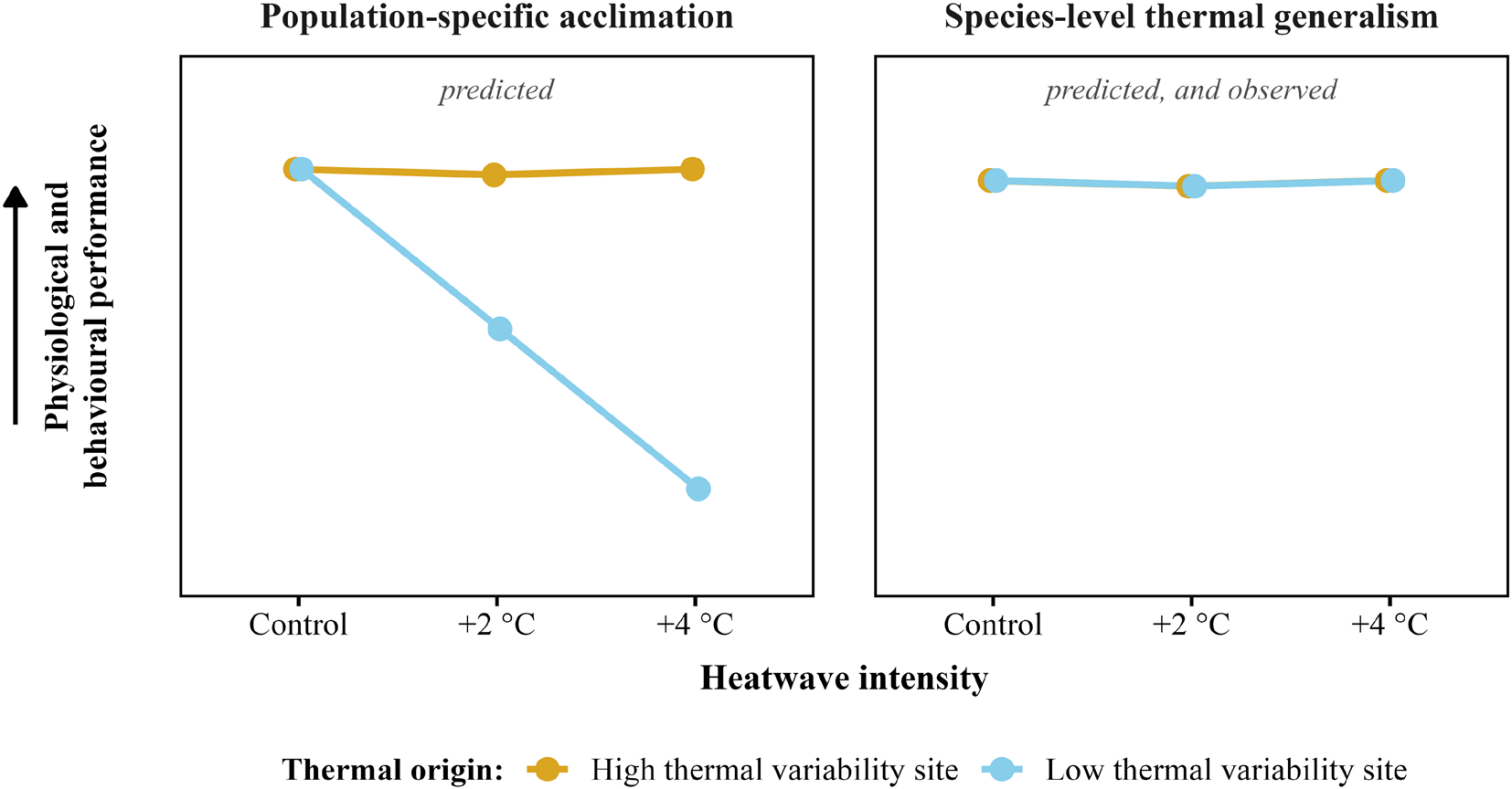
Conceptual predictions for estuarine fish performance under marine heatwaves of increasing intensity, contrasting the two competing hypotheses tested in this study. Under population‐specific acclimation (left), fish from low‐variability sites (blue) would decline in performance as heatwave intensity increases, while fish from high‐variability sites (gold) would be buffered by prior exposure to thermal extremes. Under species‐level thermal generalism (right), both groups would maintain performance irrespective of thermal origin. Lines and points represent conceptual predictions rather than measured values; performance is shown on an arbitrary scale. Our field and experimental results were consistent with the thermal generalism prediction, indicating that broad thermal tolerance in these species reflects evolutionary history in variable coastal environments rather than local acclimation.

The sole exception to the *climate variability hypothesis* operating at the species level was found for oxidative damage in one species. We observed a significant effect of thermal history on oxidative damage in *R. sarba* , where fish from low temperature variability sites had higher oxidative damage levels than those from high temperature variability sites. However, this pattern was not replicated in either *S. ciliata* or *A. australis* and should be interpreted cautiously given the limited sample size for *R. sarba* and the small number of latitudes at which both site types co‐occurred for this species. Moreover, the direction of the effect, higher oxidative damage in fish from thermally more stable environments, is inconsistent with a stress‐driven interpretation. Fish from high‐variability sites may undergo more rapid membrane lipid remodelling in response to fluctuating temperatures, increasing membrane saturation and reducing susceptibility to lipid peroxidation relative to fish from thermally stable environments where higher proportions of unsaturated membrane lipids may persist (Missionário et al. 2024). Alternatively, the pattern may reflect stochastic individual variation or unmeasured differences in diet between estuaries as opposed to a systematic effect of climate variability on cellular redox balance (Madeira et al. 2026). Despite this exception, our overarching findings provide consistent support for the *thermal generalism hypothesis* and the species‐level predictions of the *climate variability hypothesis* (Janzen 1967; Addo‐Bediako et al. 2000). We acknowledge, however, that our observational design cannot establish whether the tolerance we observed reflects active maintenance shaped by chronic thermal exposure or constitutive thermal generalism independent of local conditions; distinguishing these mechanisms would require common‐garden experiments or transcriptomic approaches capable of detecting signatures of local thermal adaptation. We also note that the experimental component was conducted using fish from a single high‐ and low‐variability population per species, which limits the generality of conclusions drawn from the laboratory data alone. Heatwave responses may differ across latitudes given that populations at range margins may experience different selective pressures and thermal histories than those sampled here, and replication across additional populations spanning the full latitudinal range would strengthen confidence in the thermal generalism interpretation. While inter‐estuary movement cannot be entirely excluded as a factor influencing population thermal history, the closed‐mouth status of Narrabeen Lagoon during our study period limits this possibility for our high‐variability site, and strong within‐estuary site fidelity reported for *A. australis* (Gunton et al. 2025) suggests that fish are unlikely to have recently immigrated from thermally contrasting environments prior to collection. The absence of population‐level differences in thermal performance across our latitudinal gradient is also consistent with high gene flow and limited genetic structuring expected in broadly mobile estuarine species (Gunton et al. 2025). Future studies incorporating acoustic telemetry, thermal logger deployments and population genomic approaches would allow individual thermal histories and adaptive genetic variation to be examined directly, providing a more precise test of whether thermal generalism reflects shared ancestral tolerance or ongoing gene flow preventing local thermal adaptation. Our findings contribute to the growing body of evidence that broadly distributed estuarine fishes—and other taxa inhabiting wide thermal ranges across broad spatial scales—can be tolerant to climatic extremes and chronic climate change stressors (Coleman et al. 2019; Sunday et al. 2014). Economically important estuarine fishes appear broadly tolerant of climate extremes, likely underpinned by thermal generalism shaped through long evolutionary exposure to variable coastal environments, with positive implications for the fisheries they support under climate change.

Some fish showed a positive response to higher baseline (i.e., not heatwave) thermal conditions both in the field and in the laboratory. We found that growth rates were higher in *S. ciliata* collected from high‐ than low‐climate variability sites under heatwave control temperatures, and fish from high‐climate variability sites also showed a steeper allometric scaling relationship in the field, with greater mass accumulating per increase in body length. While the growth difference under experimental conditions partly reflects the intentionally warmer origin‐specific experimental baseline temperature for HVS fish, the field‐based allometric pattern is independent of this design feature and suggests that *S. ciliata* may genuinely benefit from the warmer temperatures characteristic of high‐variability sites, irrespective of latitude. While our 7–14‐day heatwave trials capture short‐term energetic allocation rather than long‐term growth trajectories, our findings are supported by longer‐term experimental evidence demonstrating enhanced growth in *Sillago* spp. under elevated temperatures (Tan et al. 2024; Shalders et al. 2026), providing confidence that the patterns observed here reflect genuine thermal responses rather than artefacts of trial duration. The temperatures recorded at HVS estuary sites, frequently exceeding 30°C and occasionally approaching 37°C, fall well above the reported preferred temperature range for *S. ciliata* (21.2°C–27.4°C; Froese and Pauly 2020), yet fish from HVS sites showed no evidence of physiological stress and appeared to allocate more energy to somatic growth. Combined, our results suggest that for *S. ciliata* , thermal generalism may encompass not only tolerance of extremes but a capacity to capitalise on warmer conditions—a distinction with important implications for predicting species responses under progressive ocean warming, and a distinction that cautions against the assumption that warming will uniformly constrain estuarine fish performance.

Our simulated heatwave experiment did not provide support for the event‐profile hypothesis. Physiological and behavioural performance of our study species ( *A. australis* and *S. ciliata* ) from either HVS or LVS estuaries did not differ between extreme (+4°C) and moderate (+2°C) heatwave treatments. Across all 12 measured metrics, including energy storage, total protein content, cellular defence, oxidative damage, growth, activity, boldness, foraging rates and escape responses, fish exposed to a seven‐day +4°C heatwave responded similarly to fish experiencing control and 14‐day +2°C heatwave conditions, despite equivalent cumulative thermal exposure and irrespective of their thermal history. Our findings align with evidence that short‐term exposure to elevated temperatures does not necessarily incur immediate physiological or behavioural costs in fishes when temperatures remain below species‐specific maximum tolerance limits (Beitinger et al. 2000; Claireaux and Lefrançois 2007). The absolute temperatures achieved during our most extreme heatwave treatment (+4°C; 28°C–30°C) were well below the maximum field temperatures routinely experienced by fish at the sampled estuaries (> 35°C), suggesting that our experimental warming, while ecologically realistic in terms of rate and duration, did not approach the thermal limits these populations already tolerate in the wild. While our study did not include direct measures of absolute thermal limits such as CT_max_, our field temperature data provide ecologically realistic evidence of broad thermal tolerance. Fish at high thermal variability estuaries routinely experienced temperatures of 36°C–37°C (Table S2), conditions that would approach or exceed reported CT_max_ values for most temperate taxa (Verberk et al. 2026), yet maintained comparable physiological performance to conspecifics from thermally stable environments. The persistence and performance maintenance of these populations under field temperatures that already exceed end‐of‐century climate projections provides direct ecological evidence of broad thermal tolerance that laboratory ramping protocols cannot replicate, as no controlled experiment can expose fish to the full thermal regime these populations naturally experience in the wild. Although the +4°C treatment exceeded reported upper *preferred* temperatures for both species ( *S. ciliata* : 27.4°C; *A. australis* : 26.7°C; Froese and Pauly 2020), estuarine fishes often maintain or improve physiological performance under elevated temperatures (Morrongiello et al. 2014; Payne et al. 2016), and enhanced growth in both *A. australis* and *Sillago* spp. (Curley et al. 2013; Coleman et al. 2019; Tan et al. 2024) and associated behavioural changes under warming (O'Connor and Booth 2021) have been reported elsewhere. The maintenance of physiological and behavioural performance across both heatwave intensities is consistent with the broad thermal tolerance expected of these species and supports the view that acute heatwave events of the intensity and duration projected under near‐future climate scenarios are unlikely to impose meaningful short‐term costs on estuarine fishes possessing thermal generalism.

The maintenance of performance across all behavioural and physiological proxies, regardless of heatwave intensity and duration, has broader implications for how biological vulnerability to marine heatwaves is conceptualised. Predictive frameworks commonly emphasise event intensity as a primary driver of ecological impact (Smale et al. 2019; Oliver et al. 2021), yet our findings suggest that for thermally generalist taxa, the intensity–duration structure of a heatwave may matter less than where peak temperatures fall relative to species‐specific tolerance limits. Both heatwave profiles tested here fell within the apparent tolerance envelope of our study species, and we suggest that exceeding species‐specific thermal thresholds, rather than event profile per se, may be the more meaningful determinant of physiological and behavioural costs in thermal generalists. This distinction becomes increasingly relevant as climate projections point toward heatwaves that are more prolonged, more frequent and more intense (Frölicher et al. 2018; Oliver et al. 2018). Our results suggest that intensity‐driven vulnerability frameworks may not generalise to thermally generalist taxa, highlighting the need to incorporate species‐level thermal generalism into predictions of heatwave impacts on aquatic communities, particularly for estuarine species whose evolutionary history in variable environments may already have equipped them for the thermal extremes projected under climate change. Furthermore, fish as a taxonomic group possess relatively high aerobic scope and mobility compared to many other aquatic organisms, conferring greater capacity for behavioural and physiological flexibility in response to thermal extremes (Pörtner and Farrell 2008; Johansen et al. 2024). This functional capacity may further buffer estuarine fishes against heatwave conditions that prove damaging to less mobile or more metabolically constrained taxa such as corals, seagrasses and benthic invertebrates, thus highlighting the importance of incorporating functional group differences into climate vulnerability frameworks.

## 5 Conclusions

We show that three economically important estuarine fishes exhibit broad thermal generalism. All three species ( *Acanthopagrus australis* , *Sillago ciliata* and *Rhabdosargus sarba* ) largely maintained physiological performance across climatic extremes regardless of local thermal history or heatwave exposure. Extreme heatwave tolerance was confirmed experimentally— *A. australis* and *S. ciliata* maintained behavioural and physiological performance across moderate and extreme heatwave treatments independent of their thermal histories. The consistency of this pattern across species, climate regions and experimental conditions points toward species‐level thermal generalism, likely rooted in the long evolutionary history of estuarine taxa in chronically variable coastal environments, as the primary driver of their climatic tolerance, rather than population‐specific acclimatisation to local thermal regimes. Therefore, we conclude that the thermal generalism of estuarine fishes and the broad climatic tolerance it confers position these species and the fisheries they support to withstand intensifying climatic extremes into the near future.

## Author Contributions

**Angus Mitchell:** conceptualization, methodology, formal analysis, data curation, visualization, investigation, writing – original draft, writing – review and editing. **David J. Booth:** resources, conceptualization, writing – review and editing, funding acquisition. **Sean D. Connell:** conceptualization, funding acquisition, writing – review and editing. **Timothy Ravasi:** funding acquisition, writing – review and editing. **Ivan Nagelkerken:** conceptualization, investigation, funding acquisition, writing – review and editing, methodology, supervision, resources, project administration.

## Funding

This work was supported by the Australian Research Council, DP230101932.

## Ethics Statement

All data were collected under University of Adelaide animal ethics approval S‐2023‐043 and followed the university's animal ethics guidelines. Fish were collected under a NSW Department of Primary Industries scientific collection permit (Section 37; permit no. FP22/21) held by D.J.B.

## Conflicts of Interest

The authors declare no conflicts of interest.

## Supporting information


**Table S1:** Mean in situ abiotic conditions measured at estuaries along NSW coastline Australia during January and March 2024. Values are means ± standard error of water temperature (°C), pH (NBS scale), dissolved oxygen (DO; mg L^−1^) and salinity (PSU) recorded at the time of sampling. Sites were classified as high (HVS) or low (LVS) climate variability sites. *No fish were collected at the Iluka Bay site during the March 2024 sampling period.
**Table S2:** Summary of continuous temperature logger data across estuarine sites classified as high‐variability (HVS) or low‐variability (LVS). For each site and sampling period (January and March 2024), we report total observation time (minutes), mean temperature, standard deviation (SD), minimum and maximum temperatures and total temperature range derived from all available logger observations. Logger deployment duration varied among sites and months due to logistical constraints; metrics were therefore calculated using all observations per site within each sampling period to characterise short‐term thermal exposure and variability rather than to standardise sampling effort across sites.
**Table S3:** Sample sizes for field‐collected physiological proxies measured in three estuarine fish species across a 1000‐km latitudinal gradient. Values indicate the number of individual fish (N fish) and estuaries (N estuaries) contributing to each proxy for each species. Length weight scaling analyses were based on all individuals measured for total length and wet weight, whereas biochemical proxies were measured on a subset of individuals. Mean total length and mean wet weight (±SD) are reported next to total *N* fish collected for each species.
**Table S4:** MHW characteristics for the Sydney bioregion between 1982 and 2024, and targeted simulated MHW profiles for the heatwave experiment.
**Table S5:** Replication matrix at the commencement of the simulated marine heatwave experiment showing the number of fish (*N* = 10) used for each species ( *Acanthopagrus australis* and *Sillago ciliata* ), site origin (LVS = low variability site, HVS = high variability site) and temperature treatment (Control, MHW 1 [+2°C], MHW 2 [+4°C]). Each treatment was run for a total experimental period of 15 days (+4°C MHW) or 20 days (+2°C MHW), inclusive of a 5‐day holding period at control temperature, a ramping period of +1°C·day^−1^, and the full heatwave exposure duration (7 and 14 days respectively), with the day of reaching maximum temperature counted as Day 1 of exposure.
**Table S6:** Summary statistics of water temperature recorded by in situ loggers at high‐ (HVS) and low‐variability (LVS) estuarine sites. Values represent logger‐level means calculated over the deployment period.
**Table S7:** Mean (± SE) physiological proxies for three estuarine fish species collected across a ~1000 km latitudinal gradient from high‐climate variability (HVS; orange) and low‐climate variability (LVS; blue) estuarine sites. TP = total protein; TAC = total antioxidant capacity; MDA = oxidative damage (malondialdehyde). — indicates not sampled at that latitude or SE not calculable (*n* < 2).
**Table S8:** Fixed‐effect coefficients from linear mixed‐effects models (glmmTMB, Gaussian family) testing the effects of log‐transformed total length (log[TL]) and climate condition (LVS vs. HVS; high‐variability sites as reference) on physiological proxies in three estuarine fish species collected across a ~1000‐km latitudinal gradient. Length–weight models include a log(TL) × Climate condition interaction to test for differences in allometric scaling between site types. All models include estuary system as a random intercept (1 | Estuary system). β = coefficient estimate (natural‐log scale); SE = standard error; R2m = marginal R2 (fixed effects only); R2c = conditional R2 (fixed + random effects). Significant effects (*p* < 0.05) are highlighted.
**Table S9:** Results of generalised linear models (GLMs) testing physiological proxies in Yellowfin Bream ( *Acanthopagrus australis* ) exposed to marine heatwave (MHW) treatments and origins (low‐ vs. high‐variability sites). For each proxy, coefficients, Type III ANOVA likelihood ratio chi‐square (LR Chisq) values, *p*‐values and model explanatory power (*R* ^2^ and adjusted *R* ^2^) are reported. The delta length–weight scaling model is summarised with *F*‐tests and variance explained (*R* ^2^/adjusted *R* ^2^). Bold *p*‐values denote statistical significance (*p* < 0.05).
**Table S10:** Results of generalised linear models (GLMs) testing physiological proxies in Sand Whiting ( *Sillago ciliata* ) exposed to marine heatwave (MHW) treatments and origins (low‐ vs. high‐variability sites). For each proxy, coefficients, Type III ANOVA likelihood ratio chi square (LR Chisq) values, *p*‐values and model explanatory power (*R* ^2^ and adjusted *R* ^2^) are reported. Pairwise post hoc Tukey tests are provided for total length growth, showing comparisons among MHW levels within each origin and between origins within each MHW level. The delta length–weight scaling model is summarised with *F*‐tests and variance explained (*R* ^2^/adjusted *R* ^2^). Bold *p*‐values denote statistical significance (*p* < 0.05).
**Table S11:** Results of generalised linear models testing behavioural responses of Yellowfin Bream ( *Acanthopagrus australis* ) and Sand Whiting ( *Sillago ciliata* ) to marine heatwave (MHW) treatments and origin (low vs. high variability sites). For each behaviour, main effects and interaction terms are reported with likelihood ratio chi‐square values (LR Chisq) and associated *p*‐values. Non‐significant results (*p* ≥ 0.05) are denoted ‘ns’. Model explanatory power is shown as *R* ^2^/adjusted *R* ^2^.
**Table S12:** Number of mortalities recorded during the simulated marine heatwave experiment for each species, origin (LVS = low‐variability site; HVS = high‐variability site) and heatwave treatment. Seven mortalities were recorded in total (~6% of fish used). — indicates zero mortalities for specific treatment. No mortalities occurred in either species under the +4°C MHW treatment.
**Figure S1:** Physiological responses of two estuarine fishes to simulated marine heatwaves. Boxplots show (a–b) total protein content, (c–d) oxidative damage (MDA), (e–f) total antioxidant capacity (TAC) and (g–h) hepatosomatic index (HSI) in *Acanthopagrus australis* (Bream; left panels) and *Sillago ciliata* (Whiting; right panels). Fish were sourced from low‐variability sites (LVS, blue) and high‐variability sites (HVS, orange) and exposed to control (24°C LVS, 26°C HVS), +2°C, or +4°C heatwave treatments. Boxplots represent medians and interquartile ranges, with raw data points overlaid. No significant effects of treatment, origin, or their interaction were detected for any biomarker (Type III ANOVA, *p* > 0.05).
**Figure S2:** Behavioural responses of *Acanthopagrus australis* (yellowfin bream) to marine heatwave (MHW) treatments across origins (LVS and HVS). Panels show (a) feeding initiation time (s), (b) activity levels (%), (c) sheltering levels (%), (d) bite rate (bites min^−1^) and (e) flight initiation distance (cm). Treatments represent control (24°C for LVS; 26°C for HVS), MHW 1 (+2°C) and MHW 2 (+4°C). Boxplots show medians (horizontal line), interquartile range (box) and data range (whiskers), with individual fish plotted as points. Colours represent origin: LVS (light blue) and HVS (orange). NS above panels indicates no significant effect of treatment, origin, or their interaction (Type III ANOVA, *p* > 0.05).
**Figure S3:** Behavioural responses of *Sillago ciliata* (sand whiting) to marine heatwave (MHW) treatments across origins (LVS and HVS). Panels show (a) feeding initiation time (s), (b) activity levels (%), (c) sheltering levels (%), (d) bite rate (bites min^−1^) and (e) flight initiation distance (cm). Treatments represent control (24°C for LVS; 26°C for HVS), MHW 1 (+2°C) and MHW 2 (+4°C). Boxplots show medians (horizontal line), interquartile range (box) and data range (whiskers), with individual fish plotted as points. Colours represent origin: LVS (light blue) and HVS (orange). NS above panels indicates no significant effect of treatment, origin or their interaction (Type III ANOVA, *p* > 0.05).

## Data Availability

Data and code supporting the findings of this paper were provided to reviewers and editors during the peer review process and are publicly available at Figshare: https://figshare.com/s/9c263e491c4df2e7c884.
