# Supplementary material for: Thermal Generalism Shapes Tolerance to Extreme Heatwaves in Estuarine Fishes
Source: Glob Chang Biol. 2026 Oct 19;32(10):e71143. doi: 10.1111/gcb.71143 (PMC13680845; doi:10.1111/gcb.71143)
Supplement: Supplementary file 1 [file GCB-32-e71143-s001.pdf]

Appendix S1

Table S1. Mean *in situ* abiotic conditions measured at estuaries along NSW coastline Australia during January and March 2024. Values are means ± standard error of water temperature (°C), pH (NBS scale), dissolved oxygen (DO; mg L^{-1}), and salinity (PSU) recorded at the time of sampling. Sites were classified as high (HVS) or low (LVS) climate variability sites. * No fish were collected at the Iluka Bay site during the March 2024 sampling period.

Location	Site	Climate Condition	Period	Mean temperature	Mean pH	Mean DO	Mean Salinity
Tweed River	Tweed River	LVS	Jan 2024	26.85 (± 0.12)	8.13 (± 0.01)	6.21 (± 0.10)	31.23 (± 0.20)
	Tweed River	LVS	Mar 2024	25.30 (± 0.04)	8.48 (± 0.01)	6.81 (± 0.03)	35.08 (± 0.06)
	Wommin Lagoon	HVS	Jan 2024	30.28 (± 1.26)	7.96 (± 0.01)	6.84 (± 0.37)	29.97 (± 0.23)
	Wommin Lagoon	HVS	Mar 2024	28.92 (± 0.02)	8.29 (± 0.01)	6.55 (± 0.04)	33.02 (± 0.04)
Cudgera Creek Estuary	Cudgera Creek	LVS	Jan 2024	28.54 (± 0.49)	8.21 (± 0.01)	5.34 (± 0.37)	30.03 (± 0.37)
	Cudgera Creek	LVS	Mar 2024	24.56 (± 0.02)	8.37 (± 0.01)	5.65 (± 0.03)	33.58 (± 0.04)
	Christies Creek	HVS	Jan 2024	31.71 (± 0.51)	8.03 (± 0.01)	5.24 (± 0.96)	27.89 (± 0.75)
	Christies Creek	HVS	Mar 2024	27.64 (± 0.02)	8.06 (± 0.01)	5.69 (± 0.03)	32.70 (± 0.03)
Richmond River	Prospect Lake	HVS	Jan 2024	31.30 (± 0.23)	8.01 (± 0.01)	5.34 (± 0.08)	28.70 (± 0.11)
	Prospect Lake	HVS	Mar 2024	26.22 (± 0.06)	8.05 (± 0.01)	5.29 (± 0.04)	31.38 (± 0.07)
	South Ballina Reach	LVS	Jan 2024	24.16 (± 0.09)	8.21 (± 0.01)	6.75 (± 0.04)	32.78 (± 0.20)
	South Ballina Reach	LVS	Mar 2024	25.04 (± 0.02)	8.45 (± 0.01)	7.65 (± 0.07)	35.14 (± 0.05)
Clarence River	Whiting Bay Inlet	HVS	Jan 2024	31.45 (± 0.63)	8.21 (± 0.01)	7.92 (± 0.35)	30.43 (± 0.08)
	Whiting Bay Inlet	HVS	Mar 2024	25.54 (± 0.04)	8.27 (± 0.01)	6.35 (± 0.02)	32.26 (± 0.08)
	Iluka Bay	LVS	Jan 2024	25.43 (± 0.06)	7.98 (± 0.01)	5.81 (± 0.04)	30.22 (± 0.09)
	Iluka Bay*	LVS	Mar 2024	22.66 (± 0.02)	8.39 (± 0.00)	1.80 (± 0.02)	34.66 (± 0.14)
Nambucca River	Nambucca River	HVS	Jan 2024	24.66 (± 0.05)	8.23 (± 0.01)	7.83 (± 0.04)	32.70 (± 0.03)
	Nambucca River	HVS	Mar 2024	27.82 (± 0.02)	8.41 (± 0.00)	7.61 (± 0.05)	32.64 (± 0.05)
Valla	Deep Creek	LVS	Mar 2024	25.76 (± 0.24)	8.24 (± 0.01)	6.08 (± 0.03)	32.62 (± 0.06)
South West Rocks	Back Creek	LVS	Jan 2024	24.22 (± 0.18)	8.19 (± 0.01)	7.83 (± 0.07)	32.52 (± 0.11)
	Back Creek	LVS	Mar 2024	26.52 (± 0.04)	8.41 (± 0.01)	7.52 (± 0.02)	33.36 (± 0.08)

Sydney	Careel Bay	LVS	Mar 2024	24.23 (± 0.50)	8.26 (± 0.02)	5.89 (± 0.24)	35.45 (± 0.23)
	Narrabeen Lagoon	HVS	Mar 2024	28.57 (± 0.07)	8.31 (± 0.00)	6.63 (± 0.03)	31.65 (± 0.12)
Illawarra	Lake Illawarra	LVS	Mar 2024	22.92 (± 0.08)	8.19 (± 0.00)	7.23 (± 0.01)	35.80 (± 0.25)
	Minnamurra River	LVS	Mar 2024	23.18 (± 0.16)	8.07 (± 0.00)	6.64 (± 0.02)	35.38 (± 0.20)
Bermagui River	Rose Bay	HVS	Mar 2024	25.12 (± 0.04)	8.16 (± 0.01)	6.75 (± 0.04)	36.66 (± 0.17)
	Bermagui River	LVS	Mar 2024	20.62 (± 0.02)	8.33 (± 0.00)	6.26 (± 0.02)	38.02 (± 0.14)
Merimbula	Back Lagoon	LVS	Mar 2024	22.70 (± 0.23)	7.84 (± 0.01)	5.62 (± 0.17)	33.09 (± 0.33)
	Merimbula Lake	HVS	Mar 2024	25.24 (± 0.12)	8.41 (± 0.01)	7.46 (± 0.04)	36.80 (± 0.14)
	Pambula Lake	LVS	Mar 2024	20.58 (± 0.17)	7.96 (± 0.00)	4.63 (± 0.02)	37.00 (± 0.08)

Table S2. Summary of continuous temperature logger data across estuarine sites classified as high-variability (HVS) or low-variability (LVS). For each site and sampling period (January and March 2024), we report total observation time (minutes), mean temperature, standard deviation (SD), minimum and maximum temperatures, and total temperature range derived from all available logger observations. Logger deployment duration varied among sites and months due to logistical constraints; metrics were therefore calculated using all observations per site within each sampling period to characterise short-term thermal exposure and variability rather than to standardise sampling effort across sites.

**Month**	**Site**	**Variability**	**Observation time (minutes)**	**Mean (°C)**	**SD (°C)**	**Min (°C)**	**Max (°C)**	**Temperature Range (°C)**
January	Back Creek	LVS	1858	25.287	2.042	21.56	29.77	8.21
January	Iluka Bay	LVS	1370	26.201	1.434	23.14	30.12	6.98
January	Prospect Lake	HVS	3008	27.697	3.608	22.35	36.47	14.12
January	South Ballina	LVS	236	25.271	1.064	23.83	27.41	3.58
January	Stuart Island	HVS	1653	28.419	3.391	23.62	36.47	12.85
January	Tweed River	LVS	1473	25.705	1.174	23.38	28.02	4.64
January	Whiting Bay Inlet	HVS	1640	29.663	4.210	23.30	37.81	14.51
March	Back Creek	LVS	49	26.471	0.183	25.77	26.77	1.00
March	Back Lagoon	LVS	61	22.852	0.192	22.58	23.69	1.11

March	Bermagui River Entrance	LVS	70	20.430	0.080	20.32	20.58	0.26
March	Careel Bay	LVS	51	22.445	0.308	21.91	23.98	2.07
March	Christies Creek	HVS	77	26.881	0.334	26.13	27.92	1.79
March	Cudgera Creek	LVS	27	24.694	0.375	23.33	24.97	1.64
March	Deep Creek	LVS	89	25.811	0.646	24.05	26.89	2.84
March	Iluka Bay	LVS	104	23.243	0.385	22.51	24.17	1.66
March	Lake Illawarra	LVS	67	22.700	0.072	22.44	22.82	0.38
March	Merimbula Lake	HVS	169	25.724	0.936	23.54	26.74	3.20
March	Minnamurra River	LVS	124	23.155	0.522	22.49	24.24	1.75
March	Narrabeen Lagoon	HVS	275	25.382	2.728	20.67	29.74	9.07
March	Pambula Lake	LVS	92	21.906	0.493	19.58	22.27	2.69
March	Prospect Lake	HVS	45	26.453	0.159	25.99	26.65	0.66
March	Rose Bay	HVS	41	25.374	0.198	25.23	26.01	0.78
March	South Ballina	LVS	30	25.004	0.077	24.80	25.11	0.31
March	Stuart Island	HVS	50	28.134	0.224	27.90	28.59	0.69
March	Tweed River	LVS	52	25.564	0.277	24.39	25.82	1.43
March	Whiting Bay Inlet	HVS	33	24.658	0.227	24.15	24.99	0.84
March	Wommin Lagoon	HVS	241	29.496	2.340	26.13	34.68	8.55

Table S3: Sample sizes for field-collected physiological proxies measured in three estuarine fish species across a 1000-km latitudinal gradient. Values indicate the number of individual fish (*N* fish) and estuaries (*N* estuaries) contributing to each proxy for each species. Length–weight scaling analyses were based on all individuals measured for total length and wet weight, whereas biochemical proxies were measured on a subset of individuals. Mean total length and mean wet weight (±SD) are reported next to total N fish collected for each species.

Proxy	***Acanthopagrus australis***		***Sillago ciliata***		***Rhabdosargus sarba***	
	N fish	N estuaries	N fish	N estuaries	N fish	N estuaries

Length–weight scaling	168 (TL: 77.80 ± 35.30; WW: 14.70 ± 39.34)	9	351 (TL: 57.34 ± 21.86; WW: 2.34 ± 3.92)	14	44 (TL: 68.73 ± 12.94; WW: 6.09 ± 3.27)	6
Total protein content	89	9	168	14	37	6
Total antioxidant capacity	89	9	168	14	37	6
Oxidative damage	89	9	168	14	37	6

Table S4: MHW characteristics for the Sydney bioregion between 1982 and 2024, and targeted simulated MHW profiles for the heatwave experiment.

Marine Heatwave	Duration of event (days)	Mean intensity (°C)	Max intensity (°C)	Cumulative Intensity
**Regional average**	14.21 (±1.30)	1.84 (±0.17)	2.35 (±0.21)	27.78 (±2.54)
**MHW 1**	14	2.00	2.00	28
**MHW 2**	7	4.00	4.00	28

Table S5: Replication matrix at the commencement of the simulated marine heatwave experiment showing the number of fish ($N = 10$) used for each species (*Acanthopagrus australis* and *Sillago ciliata*), site origin (LVS = low variability site, HVS = high variability site), and temperature treatment (Control, MHW 1 [+2 °C], MHW 2 [+4 °C]). Each treatment was run for a total experimental period of 15 days (+4 °C MHW) or 20 days (+2 °C MHW), inclusive of a 5-day holding period at control temperature, a ramping period of +1 $°C \cdot day^{-1}$, and the full heatwave exposure duration (7 and 14 days respectively), with the day of reaching maximum temperature counted as day 1 of exposure.

Species	Origin	Control	MHW 1 (+2 °C)	MHW 2 (+4 °C)
Acanthopagrus australis	LVS	10	10	10
	HVS	10	10	10
Sillago ciliata	LVS	10	10	10
	HVS	10	10	10

Table S6: Summary statistics of water temperature recorded by in situ loggers at high- (HVS) and low-variability (LVS) estuarine sites. Values represent logger-level means calculated over the deployment period.

Site	Logger Name	Mean temperature (°C)	SD temperature (°C)	Mean daily thermal range (°C)	Mean hourly rate of temperature change (°C h^{-1})
HVS	Narrabeen 1	20.81	2.08	6.77	0.65
HVS	Narrabeen 2	20.93	1.91	6.18	0.59
LVS	Careel 1	21.08	0.67	1.7	0.14
LVS	Careel 2	21.29	0.71	1.92	0.14

Table S7. Mean (± SE) physiological proxies for three estuarine fish species collected across a ~1,000 km latitudinal gradient from high-climate variability (HVS; orange) and low-climate variability (LVS; blue) estuarine sites. TP = total protein; TAC = total antioxidant capacity; MDA = oxidative damage (malondialdehyde). — indicates not sampled at that latitude or SE not calculable (n < 2).

Species	Latitude (°S)	Total Length (mm)		Wet Weight (g)		Total Protein (g/L)		Antioxidant Capacity (U/mg protein)		Oxidative Damage (nmol/mg protein)	
		HVS	LVS	HVS	LVS	HVS	LVS	HVS	LVS	HVS	LVS
A. australis	28.2	78.8 (±6.7)	212.2 (±37.0)	10.83 (±2.83)	194.98 (±78.94)	1.990 (±0.111)	1.943 (±0.178)	1.627 (±0.379)	1.155 (±0.420)	1.081 (±0.129)	1.208 (±0.310)
	28.4	59.8 (±2.7)	—	4.31 (±0.67)	—	2.109 (±0.142)	—	1.552 (±0.472)	—	0.932 (±0.157)	—
	28.8	77.4 (±8.2)	142.0	9.31 (±2.94)	44.93	2.102 (±0.099)	—	0.624 (±0.074)	—	1.125 (±0.213)	—
	29.4	67.6 (±6.4)	93.3 (±7.3)	7.86 (±2.37)	20.79 (±8.47)	1.812 (±0.081)	1.925 (±0.076)	0.689 (±0.082)	0.780 (±0.098)	1.201 (±0.187)	0.913 (±0.187)
	30.6	75.5 (±4.4)	—	10.69 (±2.39)	—	1.883 (±0.097)	—	0.871 (±0.175)	—	1.454 (±0.336)	—
	30.9	—	65.3 (±2.8)	—	5.44 (±0.64)	—	1.771 (±0.076)	—	1.019 (±0.144)	—	1.123 (±0.242)
	33.6	—	68.9 (±6.4)	—	6.75 (±1.73)	—	1.913 (±0.120)	—	0.887 (±0.192)	—	0.953 (±0.298)
	33.7	113.8 (±14.5)	—	27.09 (±9.09)	—	1.931 (±0.106)	—	0.491 (±0.139)	—	0.751 (±0.150)	—
	34.5	60.5 (±2.5)	—	3.33 (±0.52)	—	1.737 (±0.169)	—	0.597 (±0.172)	—	1.338 (±0.241)	—
S. ciliata	28.2	89.8 (±26.0)	71.3 (±4.5)	11.53 (±8.96)	3.49 (±0.74)	1.821 (±0.181)	1.913 (±0.153)	1.721 (±0.236)	0.843 (±0.288)	1.444 (±0.327)	0.817 (±0.130)
	28.4	59.0 (±5.8)	51.3 (±2.7)	3.41 (±1.87)	1.37 (±0.21)	2.207 (±0.080)	2.220 (±0.101)	0.642 (±0.107)	0.855 (±0.173)	0.642 (±0.096)	1.105 (±0.185)
	28.8	53.2 (±5.4)	38.4 (±4.0)	1.63 (±0.44)	0.77 (±0.23)	2.001 (±0.092)	2.023 (±0.095)	0.773 (±0.132)	0.974 (±0.142)	0.867 (±0.132)	0.941 (±0.224)
	29.4	48.0 (±3.0)	73.3 (±3.4)	1.27 (±0.28)	3.90 (±0.41)	1.863 (±0.133)	1.999 (±0.098)	0.898 (±0.127)	1.214 (±0.286)	1.070 (±0.373)	1.025 (±0.284)
	30.6	69.1 (±2.3)	40.4 (±1.3)	3.00 (±0.29)	0.61 (±0.05)	2.138 (±0.130)	2.128 (±0.128)	1.221 (±0.322)	1.239 (±0.272)	1.400 (±0.481)	1.451 (±0.242)
	30.9	—	81.1 (±5.9)	—	5.24 (±1.24)	—	2.272 (±0.122)	—	1.091 (±0.217)	—	0.659 (±0.146)
	33.6	—	64.5 (±4.9)	—	2.86 (±0.63)	—	2.299 (±0.124)	—	0.532 (±0.105)	—	0.888 (±0.323)
	33.7	52.5 (±7.1)	—	1.73 (±0.75)	—	2.229 (±0.060)	—	0.622 (±0.202)	—	1.344 (±0.292)	—
	34.5	—	29.0 (±3.5)	—	0.41 (±0.17)	—	2.114 (±0.024)	—	0.867 (±0.210)	—	0.877 (±0.373)
	36.4	62.5 (±3.3)	51.1 (±2.3)	2.32 (±0.37)	1.43 (±0.19)	1.970 (±0.076)	1.932 (±0.111)	1.168 (±0.254)	1.310 (±0.179)	0.828 (±0.114)	1.325 (±0.215)
	36.9	50.4 (±3.8)	55.0 (±2.4)	1.39 (±0.27)	1.79 (±0.23)	1.893 (±0.094)	2.038 (±0.069)	1.065 (±0.213)	1.004 (±0.103)	1.447 (±0.260)	1.170 (±0.140)
	28.4	49.8 (±3.4)	—	2.18 (±0.41)	—	2.097 (±0.129)	—	0.863 (±0.228)	—	0.486 (±0.195)	—

Species	Latitude (°S)	Total Length (mm)		Wet Weight (g)		Total Protein (g/L)		Antioxidant Capacity (U/mg protein)		Oxidative Damage (nmol/mg protein)	
		HVS	LVS	HVS	LVS	HVS	LVS	HVS	LVS	HVS	LVS
R. sarba	**29.4**	70.0 (±6.0)	67.0	5.30 (±1.32)	4.69	1.882 (±0.358)	2.083	1.507 (±1.031)	1.280	0.729 (±0.486)	0.770
	**30.6**	75.0 (±4.1)	—	7.51 (±1.28)	—	1.884 (±0.132)	—	1.099 (±0.237)	—	0.595 (±0.128)	—
	**30.9**	—	72.8 (±3.0)	—	7.29 (±0.98)	—	1.749 (±0.054)	—	0.914 (±0.140)	—	1.353 (±0.175)
	**33.6**	—	71.2 (±1.3)	—	6.19 (±0.28)	—	2.097 (±0.138)	—	0.679 (±0.087)	—	1.261 (±0.386)
	**33.7**	95.0	—	13.47	—	2.238	—	0.715	—	0.259	—

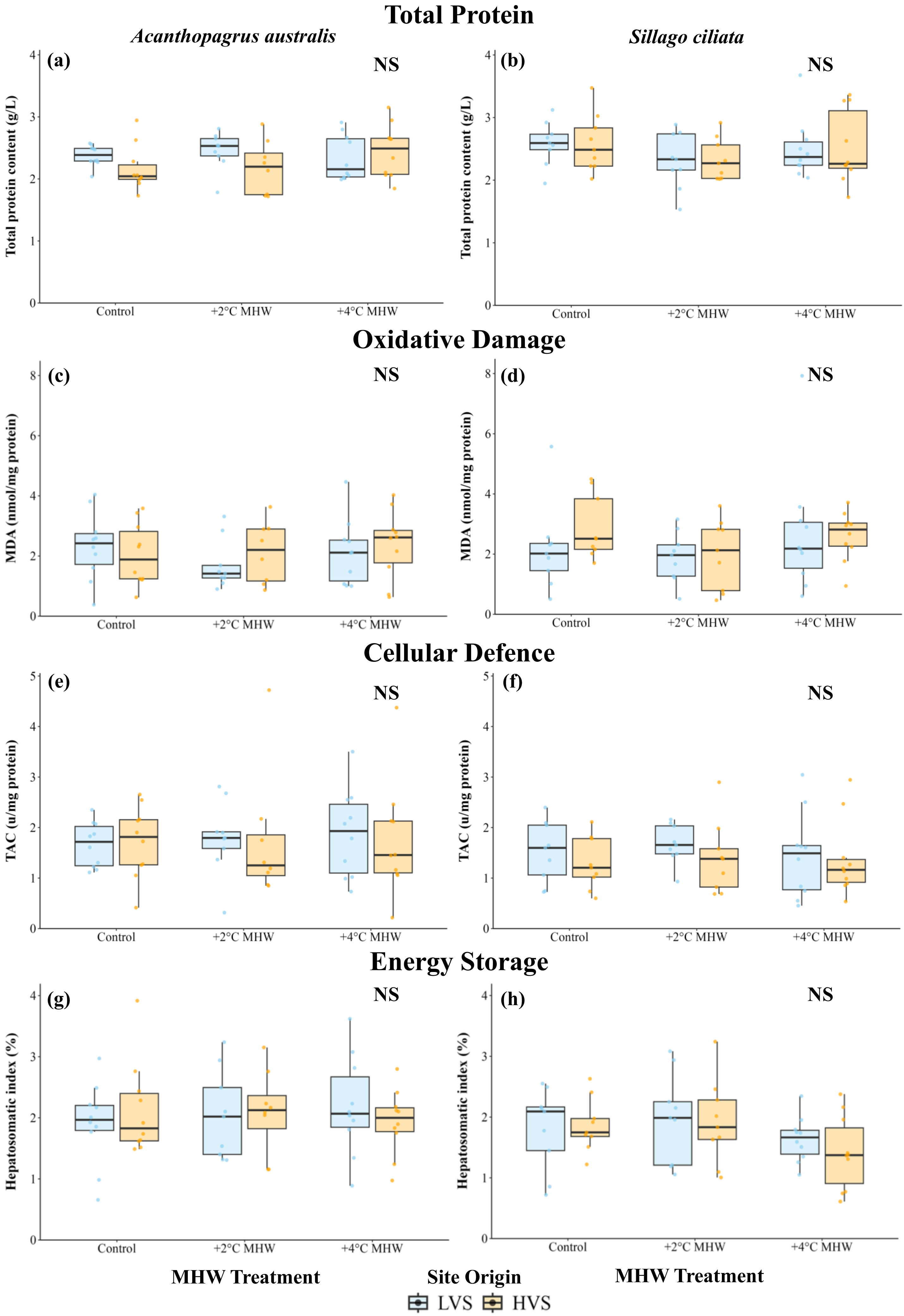

Total Protein
Acanthopagrus australis
Sillago ciliata
(a)
(b)
NS
Total protein content (g/L)
Control
+2°C MHW
+4°C MHW
Oxidative Damage
(c)
(d)
MDA (nmol/mg protein)
Cellular Defence
(e)
(f)
TAC (u/mg protein)
Energy Storage
(g)
(h)
Hepatosomatic index (%)
MHW Treatment
Site Origin
LVS
HVS

Figure S1. Physiological responses of two estuarine fishes to simulated marine heatwaves. Boxplots show (a–b) total protein content, (c–d) oxidative damage (MDA), (e–f) total antioxidant capacity (TAC), and (g–h) hepatosomatic index (HSI) in *Acanthopagrus australis* (Bream; left panels) and *Sillago ciliata* (Whiting; right panels). Fish were sourced from low-variability sites (LVS, blue) and high-variability sites (HVS, orange) and exposed to control (24 °C LVS, 26 °C HVS), +2 °C, or +4 °C heatwave treatments. Boxplots represent medians and interquartile ranges, with raw data points overlaid. No significant effects of treatment, origin, or their interaction were detected for any biomarker (Type III ANOVA, $p > 0.05$).

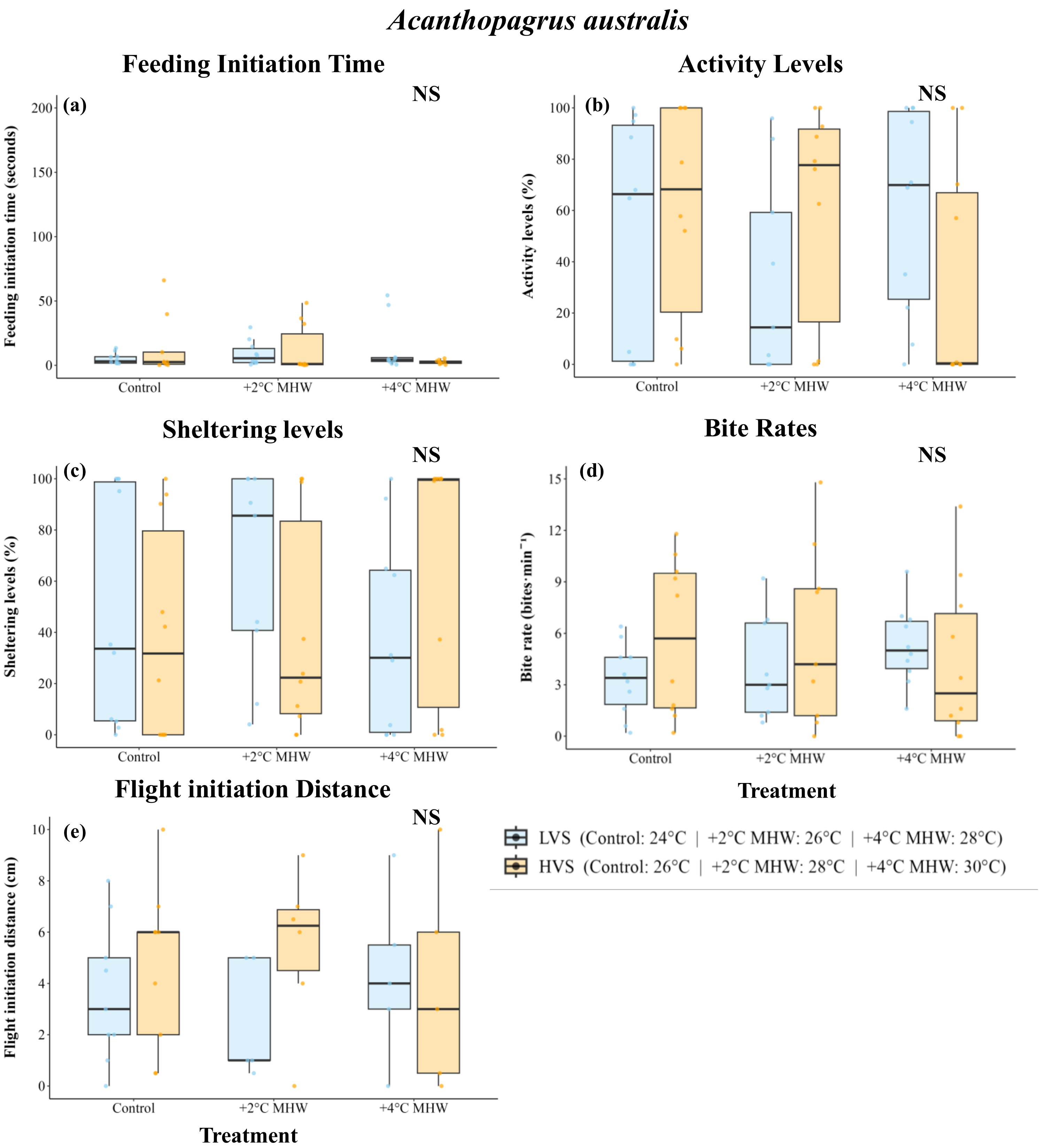

Figure S2. Behavioural responses of *Acanthopagrus australis* (yellowfin bream) to marine heatwave (MHW) treatments across origins (LVS and HVS). Panels show (a) feeding initiation time (s), (b) activity levels (%), (c) sheltering levels (%), (d) bite rate (bites min^{-1}), and (e) flight initiation distance (cm). Treatments represent control (24 °C for LVS; 26 °C for HVS),

MHW 1 (+2 °C), and MHW 2 (+4 °C). Boxplots show medians (horizontal line), interquartile range (box), and data range (whiskers), with individual fish plotted as points. Colours represent origin: LVS (light blue) and HVS (orange). NS above panels indicates no significant effect of treatment, origin, or their interaction (Type III ANOVA, $p > 0.05$).

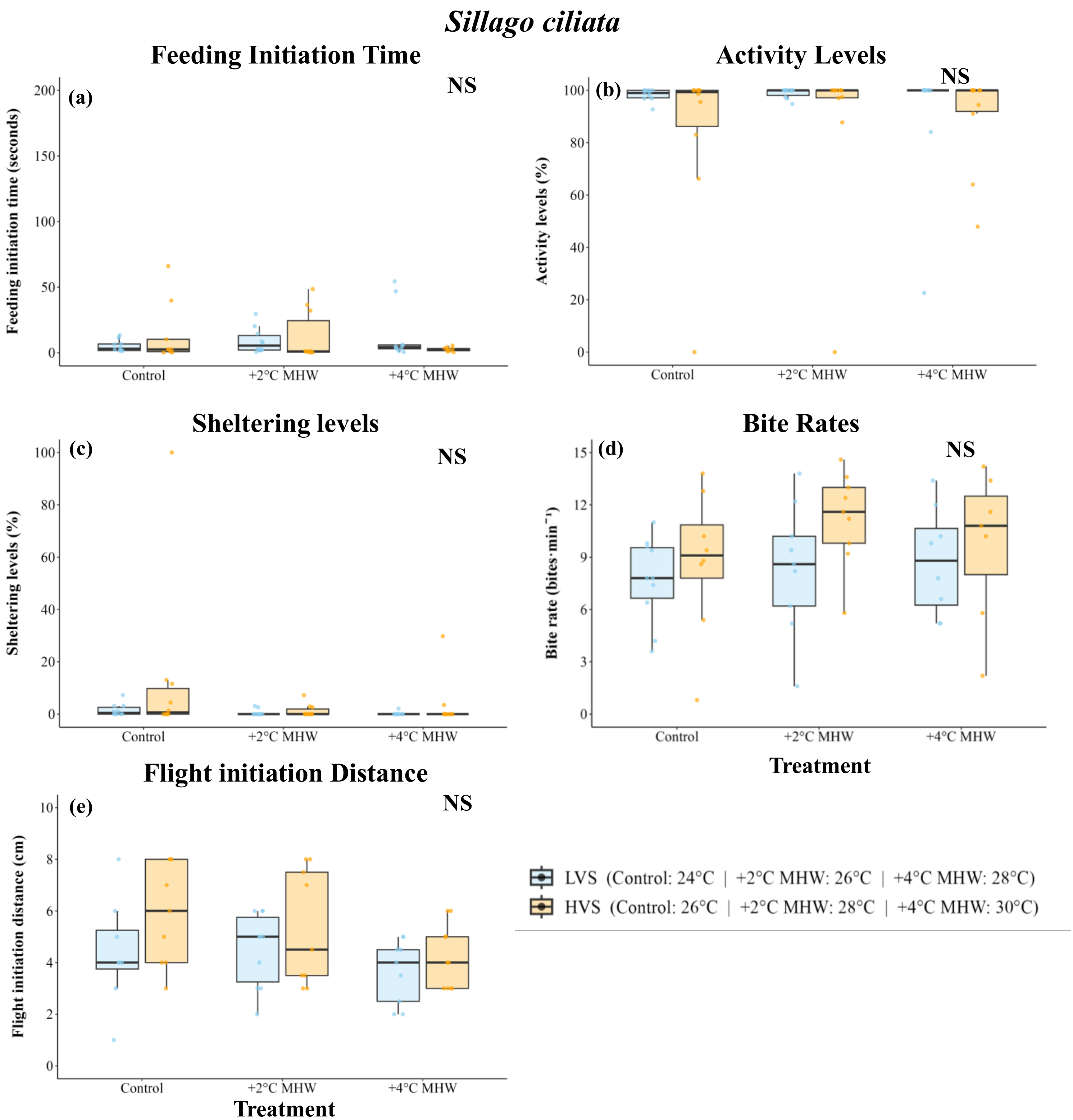

Figure S3. Behavioural responses of *Sillago ciliata* (sand whiting) to marine heatwave (MHW) treatments across origins (LVS and HVS). Panels show (a) feeding initiation time (s), (b) activity levels (%), (c) sheltering levels (%), (d) bite rate (bites min^{-1}), and (e) flight initiation distance (cm). Treatments represent control (24 °C for LVS; 26 °C for HVS), MHW 1 (+2 °C), and MHW 2 (+4 °C). Boxplots show medians (horizontal line), interquartile range

(box), and data range (whiskers), with individual fish plotted as points. Colours represent origin: LVS (light blue) and HVS (orange). NS above panels indicates no significant effect of treatment, origin, or their interaction (Type III ANOVA, $p > 0.05$).

Table S8. Fixed-effect coefficients from linear mixed-effects models (glmmTMB, Gaussian family) testing the effects of log-transformed total length (log[TL]) and climate condition (LVS vs HVS; high-variability sites as reference) on physiological proxies in three estuarine fish species collected across a ~1,000-km latitudinal gradient. Length–weight models include a log(TL) × Climate condition interaction to test for differences in allometric scaling between site types. All models include estuary system as a random intercept (1 | Estuary system). β = coefficient estimate (natural-log scale); SE = standard error; R^2m = marginal R^2 (fixed effects only); R^2c = conditional R^2 (fixed + random effects). Significant effects ($p < 0.05$) are highlighted.

Species	Proxy	n	Model structure	Fixed effect	β	SE	z	P	R²m	R²c
S. ciliata	**Length–weight**	351	log(WW) ~ log(TL) × Climate condition + (1 | Estuary system)	log(TL)	2.845	0.029	99.20	**<0.001**	0.988	0.990
				Climate condition (LVS vs HVS)	0.525	0.144	3.64	**<0.001**		
				log(TL) × Climate condition	−0.125	0.036	−3.46	**<0.001**		
	**Total protein (g/L)**	168	ln(TP + 1) ~ log(TL) + Climate condition + (1 | Estuary system)	log(TL)	0.031	0.028	1.14	0.256	0.013	0.107
				Climate condition (LVS vs HVS)	0.015	0.019	0.81	0.419		
	**Antioxidant capacity (U/mg)**	168	ln(TAC + 1) ~ log(TL) + Climate condition + (1 | Estuary system)	log(TL)	0.127	0.070	1.81	0.070	0.024	0.070
				Climate condition (LVS vs HVS)	0.036	0.048	0.76	0.447		
	**Oxidative damage (nmol/mg)**	168	ln(MDA + 1) ~ log(TL) + Climate condition + (1 | Estuary system)	log(TL)	−0.152	0.073	−2.09	**0.037**	0.026	0.026
				Climate condition (LVS vs HVS)	−0.003	0.050	−0.07	0.947		

Species	Proxy	n	Model structure	Fixed effect	β	SE	z	P	R²m	R²c
R. sarba	**Length–weight**	44	log(WW) ~ log(TL) × Climate condition + (1 | Estuary system)	log(TL)	2.833	0.109	26.00	**<0.001**	0.982	0.984
				Climate condition (LVS vs HVS)	−0.507	0.701	−0.72	0.469		
				log(TL) × Climate condition	0.134	0.165	0.81	0.418		
	**Total protein (g/L)**	37	ln(TP + 1) ~ log(TL) + Climate condition + (1 | Estuary system)	log(TL)	−0.051	0.111	−0.46	0.644	0.012	0.040
				Climate condition (LVS vs HVS)	−0.011	0.045	−0.25	0.802		
	**Antioxidant capacity (U/mg)**	37	ln(TAC + 1) ~ log(TL) + Climate condition + (1 | Estuary system)	log(TL)	−0.138	0.219	−0.63	0.528	0.046	0.046
				Climate condition (LVS vs HVS)	−0.079	0.086	−0.92	0.359		
	**Oxidative damage (nmol/mg)**	37	ln(MDA + 1) ~ log(TL) + Climate condition + (1 | Estuary system)	log(TL)	0.280	0.281	1.00	0.319	0.241	0.241
				Climate condition (LVS vs HVS)	0.309	0.111	2.80	**0.005**		
A. australis	**Length–weight**	168	log(WW) ~ log(TL) × Climate condition + (1 | Estuary system)	log(TL)	2.935	0.036	80.85	**<0.001**	0.987	0.987
				Climate condition (LVS vs HVS)	0.391	0.244	1.60	0.109		
				log(TL) × Climate condition	−0.078	0.056	−1.40	0.160		
	**Total protein (g/L)**	89	ln(TP + 1) ~ log(TL) + Climate condition + (1 | Estuary system)	log(TL)	0.026	0.032	0.80	0.425	0.022	0.022
				Climate condition (LVS vs HVS)	−0.032	0.024	−1.34	0.181		
	**Antioxidant capacity (U/mg)**	89	ln(TAC + 1) ~ log(TL) + Climate condition + (1 | Estuary system)	log(TL)	−0.058	0.099	−0.59	0.556	0.005	0.116
				Climate condition (LVS vs HVS)	0.025	0.085	0.29	0.773		
	**Oxidative damage (nmol/mg)**	89	ln(MDA + 1) ~ log(TL) + Climate condition + (1 | Estuary system)	log(TL)	−0.075	0.091	−0.83	0.409	0.016	0.016
				Climate condition (LVS vs HVS)	−0.043	0.068	−0.64	0.522		

Table S9. Results of generalized linear models (GLMs) testing physiological proxies in Yellowfin Bream (*Acanthopagrus australis*) exposed to marine heatwave (MHW) treatments and origins (low- vs. high-variability sites). For each proxy, coefficients, Type III ANOVA likelihood ratio chi-square (LR Chisq) values, *p*-values, and model explanatory power (R^2 and adjusted R^2) are reported. The delta length–weight scaling model is summarised with F-tests and variance explained (R^2 / adjusted R^2). **Bold** p-values denote statistical significance (P < 0.05).

Acanthopagrus australis				
GLM Summary – Total Protein Content: $R^2 = 0.106$, adjusted $R^2 = 0.018$				
Coefficients	Estimate	Std. Error	T value	P-value
Intercept	2.377	0.111	21.366	<0.001
Origin (HVS)	-0.211	0.157	-1.342	0.186
Treatment (MHW 1)	0.079	0.162	0.487	0.628
Treatment (MHW 2)	-0.041	0.157	-0.260	0.796
Origin (HVS) * Treatment (MHW 1)	-0.064	0.232	-0.277	0.783
Origin (HVS) * Treatment (MHW 2)	0.321	0.222	1.442	0.156
Type III ANOVA				
Factor	LR Chisq	DF	P-value	
Origin	1.803	1	0.179	
Treatment	1.168	2	0.558	
Origin * Treatment	3.291	2	0.193	
GLM Summary – Total antioxidant capacity: $R^2 = 0.007$, adjusted $R^2 = -0.091$				
Coefficients	Estimate	Std. Error	T value	P-value
Intercept	1.663	0.281	5.908	<0.001
Origin (HVS)	0.050	0.398	0.125	0.901
Treatment (MHW 1)	0.132	0.409	0.322	0.749
Treatment (MHW 2)	0.215	0.397	0.539	0.592
Origin (HVS) * Treatment (MHW 1)	-0.098	0.588	-0.167	0.868
Origin (HVS) * Treatment (MHW 2)	-0.176	0.563	-0.312	0.756
Type III ANOVA				
Factor	LR Chisq	DF	P-value	
Origin	0.031	1	0.861	
Treatment	0.209	2	0.901	
Origin * Treatment	0.098	2	0.952	
GLM Summary – Oxidative damage: $R^2 = 0.047$, adjusted $R^2 = -0.046$				
Coefficients	Estimate	Std. Error	T value	P-value
Intercept	2.329	0.331	7.046	**<0.001**
Origin (HVS)	-0.284	0.467	-0.607	0.547
Treatment (MHW 1)	-0.630	0.480	-1.312	0.195
Treatment (MHW 2)	-0.195	0.467	-0.416	0.679
Origin (HVS) * Treatment (MHW 1)	0.707	0.690	1.025	0.310
Origin (HVS) * Treatment (MHW 2)	0.529	0.661	0.800	0.427
Type III ANOVA				
Factor	LR Chisq	DF	P-value	
Origin	0.214	1	0.644	
Treatment	1.101	2	0.577	
Origin * Treatment	1.175	2	0.556	
GLM Summary – HSI: $R^2 = 0.024$, adjusted $R^2 = -0.072$				
Coefficients	Estimate	Std. Error	T value	P-value

Intercept	1.905	0.222	8.565	**<0.001**
Origin (HVS)	0.227	0.315	0.721	0.474
Treatment (MHW 1)	0.135	0.323	0.419	0.677
Treatment (MHW 2)	0.283	0.315	0.901	0.372
Origin (HVS) * Treatment (MHW 1)	-0.173	0.464	-0.372	0.711
Origin (HVS) * Treatment (MHW 2)	-0.484	0.445	-1.087	0.282

Type III ANOVA			
Factor	LR Chisq	DF	P-value
Origin	0.002	1	0.966
Treatment	0.054	2	0.973
Origin * Treatment	1.210	2	0.546
GLM Summary – Wet Weight Growth: R^2 = 0.038, adjusted R^2 = -0.056			
Type III ANOVA			
Factor	LR Chisq	DF	P-value
Origin	0.259	1	0.611
Treatment	1.430	2	0.489
Origin * Treatment	0.313	2	0.855
GLM Summary – Total Length Growth: R2 = 0.107, adjusted R2 = 0.019			
Type III ANOVA			
Factor	LR Chisq	DF	P-value
Origin	1.300	1	0.254
Treatment	4.236	2	0.120
Origin * Treatment	0.544	2	0.762

GLM Summary – Delta length – weight scaling: R^2 = 0.204, adjusted R^2 = 0.010				
Factor	Sum squares	DF	F-value	P-value
Intercept	0.026	1	6.340	**0.015**
Log10 starting length	0.025	1	6.002	**0.018**
Origin	<0.001	1	0.018	0.894
MHW	<0.001	2	0.108	0.898
Log10 starting length * Origin	<0.001	1	0.017	0.896
Log10 starting length * MHW	<0.001	2	0.119	0.889
MHW * Origin	0.006	2	0.756	0.476
Log10 starting length * MHW * Origin	0.006	2	0.778	0.465

Table S10. Results of generalized linear models (GLMs) testing physiological proxies in Sand Whiting (*Sillago ciliata*) exposed to marine heatwave (MHW) treatments and origins (low- vs. high-variability sites). For each proxy, coefficients, Type III ANOVA likelihood ratio chi-square (LR Chisq) values, *p*-values, and model explanatory power (R^2 and adjusted R^2) are reported. Pairwise post hoc Tukey tests are provided for total length growth, showing comparisons among MHW levels within each origin and between origins within each MHW level. The delta length–weight scaling model is summarised with F-tests and variance explained (R^2 / adjusted R^2). **Bold** p-values denote statistical significance (P < 0.05).

Sillago ciliata				
GLM Summary – Total Protein Content: R^2 = 0.063, adjusted R^2 = -0.029				
Coefficients	Estimate	Std. Error	T value	P-value
Intercept	2.589	0.150	17.205	**<0.001**
Origin (HVS)	<-0.001	0.213	-0.002	0.998
Treatment (MHW 1)	-0.277	0.213	-1.303	0.198
Treatment (MHW 2)	-0.093	0.207	-0.446	0.657
Origin (HVS) * Treatment (MHW 1)	0.016	0.301	0.054	0.957
Origin (HVS) * Treatment (MHW 2)	0.027	0.293	0.093	0.926
Type III ANOVA				
Factor	LR Chisq	DF	P-value	
Origin	0.014	1	0.907	
Treatment	3.394	2	0.183	
Origin * Treatment	0.009	2	0.996	
GLM Summary – Total antioxidant capacity: R^2 = 0.037, adjusted R^2 = -0.060				
Coefficients	Estimate	Std. Error	T value	P-value
Intercept	1.519	0.220	6.910	**<0.001**
Origin (HVS)	-0.230	0.311	-0.739	0.463
Treatment (MHW 1)	0.157	0.311	0.505	0.616
Treatment (MHW 2)	-0.080	0.303	-0.263	0.794
Origin (HVS) * Treatment (MHW 1)	-0.053	0.440	-0.120	0.905
Origin (HVS) * Treatment (MHW 2)	0.159	0.428	0.371	0.712
Type III ANOVA				
Factor	LR Chisq	DF	P-value	
Origin	1.213	1	0.271	
Treatment	0.480	2	0.787	
Origin * Treatment	0.267	2	0.875	
GLM Summary – Oxidative damage: R^2 = 0.079, adjusted R^2 = -0.014				
Coefficients	Estimate	Std. Error	T value	P-value

Intercept	2.186	0.438	4.995	**<0.001**
Origin (HVS)	0.691	0.619	1.116	0.270
Treatment (MHW 1)	-0.291	0.619	-0.470	0.640
Treatment (MHW 2)	0.496	0.603	0.823	0.415
Origin (HVS) * Treatment (MHW 1)	-0.582	0.875	-0.665	0.509
Origin (HVS) * Treatment (MHW 2)	-0.771	0.853	-0.904	0.370

Type III ANOVA

Factor	LR Chisq	DF	P-value
Origin	0.465	1	0.496
Treatment	2.971	2	0.226
Origin * Treatment	0.872	2	0.647

GLM Summary – HSI: $R^2 = 0.109$, adjusted $R^2 = 0.020$

Coefficients	Estimate	Std. Error	T value	P-value
Intercept	1.805	0.199	9.080	**<0.001**
Origin (HVS)	0.062	0.281	0.221	0.826
Treatment (MHW 1)	0.176	0.281	0.625	0.535
Treatment (MHW 2)	-0.171	0.274	-0.625	0.535
Origin (HVS) * Treatment (MHW 1)	-0.128	0.398	-0.322	0.749
Origin (HVS) * Treatment (MHW 2)	-0.287	0.387	-0.741	0.462

Type III ANOVA

Factor	LR Chisq	DF	P-value
Origin	0.228	1	0.633
Treatment	5.280	2	0.071
Origin * Treatment	0.553	2	0.758

GLM Summary – Wet Weight Growth: $R^2 = 0.181$, adjusted $R^2 = 0.100$

Type III ANOVA

Factor	LR Chisq	DF	P-value
Origin	2.388	1	0.122
Treatment	5.403	2	0.067
Origin * Treatment	3.427	2	0.180

GLM Summary – Total Length Growth: $R^2 = 0.182$, adjusted $R^2 = 0.100$

Type III ANOVA

Factor	LR Chisq	DF	P-value
Origin	2.455	1	0.117
Treatment	0.918	2	0.632
Origin * Treatment	7.622	2	**0.022**

Total length growth pairwise tests levels of MHW within LVS level of Origin

Contrast	Estimate	SE	DF	T ratio	P-value
Control – MHW 1	-0.216	0.097	50	-2.235	0.075
Control – MHW 2	-0.013	0.094	50	-0.142	0.989
MHW 1 – MHW 2	0.203	0.094	50	2.151	0.090

Total length growth pairwise tests levels of MHW within HVS level of Origin

Contrast	Estimate	SE	Df	T ratio	P-value
Control – MHW 1	0.143	0.097	50	1.474	0.312
Control – MHW 2	0.067	0.094	50	0.713	0.757
MHW 1 – MHW 2	-0.075	0.094	50	-0.799	0.705
Total length growth pairwise tests levels of Origin within control level of MHW					
Contrast	Estimate	SE	DF	T ratio	P-value
LVS – HVS	-0.233	0.097	50	-2.403	**0.020**
Total length growth pairwise tests levels of Origin within MHW 1 level of MHW					
Contrast	Estimate	SE	DF	T ratio	P-value
LVS – HVS	0.126	0.097	50	1.305	0.198
Total length growth pairwise tests levels of Origin within MHW 2 level of MHW					
Contrast	Estimate	SE	DF	T ratio	P-value
LVS – HVS	-0.152	0.092	50	-1.655	0.104

GLM Summary – Delta length – weight scaling: $R^2 = 0.589$, adjusted $R^2 = 0.486$				
Factor	Sum squares	DF	F-value	P-value
Intercept	0.080	1	30.812	**< 0.001**
Log10 starting length	0.079	1	30.207	**<0.001**
Origin	0.001	1	0.393	0.534
MHW	0.016	2	2.998	0.060
Log10 starting length * Origin	0.001	1	0.561	0.458
Log10 starting length * MHW	0.015	2	2.816	0.071
MHW * Origin	0.015	2	2.849	0.069
Log10 starting length * MHW * Origin	0.015	2	2.878	0.066

Table S11. Results of generalized linear models testing behavioural responses of Yellowfin Bream (*Acanthopagrus australis*) and Sand Whiting (*Sillago ciliata*) to marine heatwave (MHW) treatments and origin (low vs. high variability sites). For each behaviour, main effects and interaction terms are reported with likelihood ratio chi-square values (LR Chisq) and associated *p*-values. Non-significant results ($p \geq 0.05$) are denoted “ns”. Model explanatory power is shown as R^2 / adjusted R^2.

Species	Behaviour	Main effects (MHW, Origin, MHW×Origin)	LR Chisq (MHW)	LR Chisq (Origin)	LR Chisq (Interaction)	R^2 / Adj R^2

Yellowfin Bream	Feeding Initiation Time (s)	MHW ns, Origin ns, Interaction ns	3.30 (P=0.19)	0.86 (P=0.35)	4.02 (P=0.13)	0.15 / 0.06
	Activity levels (%)	MHW ns, Origin ns, Interaction ns	0.68 (P=0.71)	0.06 (P=0.80)	4.11 (P=0.13)	0.08 / <0.001
	Sheltering levels (%)	MHW ns, Origin ns, Interaction ns	0.46 (P=0.80)	0.04 (P=0.84)	3.42 (P=0.18)	0.07 / -0.02
	Flight Initiation Distance (cm)	MHW ns, Origin ns, Interaction ns	0.02 (P=0.99)	0.04 (P=0.84)	0.81 (P=0.67)	0.02 / -0.11
	Bite Rate (bites/min)	MHW ns, Origin ns, Interaction ns	0.45 (P=0.80)	1.96 (P=0.16)	2.77 (P=0.25)	0.09 / 0.00
Sand Whiting	Feeding Initiation Time (s)	MHW ns, Origin ns, Interaction ns	0.72 (P=0.70)	1.88 (P=0.17)	1.36 (P=0.51)	0.07 / -0.02
	Activity levels (%)	MHW ns, Origin ns, Interaction ns	0.24 (P=0.89)	2.22 (P=0.14)	0.94 (P=0.63)	0.06 / -0.03
	Sheltering levels (%)	MHW ns, Origin ns, Interaction ns	2.72 (P=0.26)	2.21 (P=0.14)	1.82 (P=0.40)	0.11 / 0.03
	Flight Initiation Distance (cm)	MHW ns, Origin ns, Interaction ns	5.23 (P=0.07)	0.49 (P=0.48)	1.09 (P=0.58)	0.11 / 0.03
	Bite Rate (bites/min)	MHW ns, Origin ns, Interaction ns	3.36 (P=0.19)	3.01 (P=0.08)	0.67 (P=0.71)	0.12 / 0.03

Table S12. Number of mortalities recorded during the simulated marine heatwave experiment for each species, origin (LVS = low-variability site; HVS = high-variability site), and heatwave treatment. Seven mortalities were recorded in total (~6% of fish used). — indicates zero mortalities for specific treatment. No mortalities occurred in either species under the +4°C MHW treatment.

Species	Origin	Control	+2°C MHW	+4°C MHW	Total
Acanthopagrus australis	LVS	1	—	—	**1**
	HVS	—	2	—	**2**
Sillago ciliata	LVS	1	1	—	**2**
	HVS	1	1	—	**2**
